# Understanding, Using, and Facilitating Evidence-Based Practice: A Scoping Review of Influencing Factors Among Nurse Managers in Acute Care

**DOI:** 10.1155/jonm/2155376

**Published:** 2025-07-04

**Authors:** Silas Yenbon Sebire, Jeremy Brown, Emmie Malewezi, Lyvonne N. Tume

**Affiliations:** ^1^Faculty of Health Social Care and Medicine, Edge Hill University, Ormskirk, UK; ^2^Department of Nursing, Presbyterian Nursing and Midwifery Training College, Dormaa Ahenkro, Ghana; ^3^Paediatric Intensive Care Unit, Alder Hey Children's Hospital, Liverpool, UK

**Keywords:** acute care, enablers and barriers, evidence-based practice, leadership, nurse managers

## Abstract

**Background:** Evidence-based practice (EBP) is essential for quality healthcare. Nurse managers (NMs) play a key role in promoting EBP adoption among nurses, given their position as clinical leaders for nurses seeking to change practice. Therefore, it is crucial to understand the factors influencing their understanding and facilitation of EBP in care delivery.

**Aim:** To synthesize literature on the enablers and barriers faced by NMs in understanding, using, and facilitating EBP within acute care settings.

**Methods:** A scoping review which followed the Joanna Briggs Institute framework for scoping reviews and reported in accordance with PRISMA extension for scoping reviews. Multiple electronic databases, including MEDLINE, CINAHL, Cochrane Library, Scopus, JBI EBP database, Embase, Emcare, HMIC, and PsycINFO regardless of publication to be comprehensive. Two independent reviewers screened studies and extracted data. Methodological quality of included studies was appraised and considered high.

**Results:** The search yielded 1338 results of which 24 studies met inclusion criteria and included in the review. Key findings are the central role of strong personal factors-such as positive attitudes, beliefs, and good EBP competencies-in driving understanding and use of EBP. Supportive leadership behaviors can create a positive organizational environment for EBP, while access to resources and infrastructure are essential for EBP implementation. Conversely, barriers such as poor EBP knowledge, skills and competency, heavy workloads, insufficient resources, and lack of effective leadership support impede EBP implementation within acute care settings.

**Conclusions:** NMs play a crucial role in EBP implementation within their organizations, emphasizing the interconnectedness of personal and organizational factors.

**Implications for Nursing management:** Developing key personal factors and specific EBP-supportive behaviors of NMs can create a powerful catalyst for overcoming barriers, leading to improved patient care outcomes.

## 1. Introduction

Evidence-based practice (EBP) underpins high-quality, safe, and effective patient care [1, 2]. It integrates current research evidence with clinical expertise and patient preferences to optimize healthcare outcomes, reduce costs, and minimize variations in care delivery [3–5]. Yet, a persistent and alarming 15 year gap exists between research generation and its implementation into nursing practice [6]. This delay necessitates action, as it hinders optimal and effective clinical care.

Nurses, who form the largest component of the healthcare workforce, and have the most direct patient contact, are key in achieving a truly evidence-based healthcare system [7–9]. EBP is now an expected competency, mandated by nursing professional bodies such as the United Kingdom (UK) Nursing and Midwifery Council (NMC) and the American Nurses Association (ANA) [10, 11]. Despite EBP integration into nursing education programs and its acknowledged importance, barriers impede its routine use in practice [12–15]. These barriers operate at individual and organizational levels, including limited resources, workload pressures, and deficiencies in EBP knowledge and skills [12, 16, 17]. Current challenges in implementing EBP in nursing highlight the critical role of nurse managers (NMs) as facilitators [18, 19].

NMs, as frontline nursing leaders, can significantly influence EBP uptake within their units and organizations. Through leadership style, resource allocation, and professional development support, they can create environments that either facilitate or hinder EBP implementation [20–23]. Positive leadership behaviors, such as fostering a supportive culture, leading by example, and providing EBP mentorship, are linked to increased EBP adoption among nurses [18, 24]. Conversely, a lack of leadership support can significantly undermine EBP efforts [19]. Despite NMs pivotal role in facilitating EBP at the bedside, evidence suggests their engagement with EBP has been passive for reasons including limited access to EBP training [25].

The growing emphasis on EBP leadership underscores the need to understand how to empower NMs to operationalize EBP within their spheres of influence [26]. Enhancing NMs' EBP capabilities is crucial for overcoming the research-practice gap and optimizing EBP implementation [26, 27]. By actively engaging NMs in EBP, healthcare organizations can accelerate the implementation of EBPs and improve the quality of patient care [4, 25, 28]. However, to effectively empower them, a better understanding of the factors that enable or hinder their understanding, use, and facilitation of EBP is needed. In this context, understanding EBP refers to NMs' cognitive grasp of EBP principles and processes, while using EBP involves applying evidence in their own decision-making and to practice. Facilitating EBP denotes their role in supporting and enabling their staff to engage with EBP. NMs in acute care operate within the demanding and complex environment of acute care with rapid patient turnover, time-sensitive decision-making requirements, and intensive resource demands that create unique barriers to EBP implementation. Nonetheless, a significant knowledge gap exists regarding these multilevel factors impacting NMs' EBP knowledge, attitudes, skills, and implementation efforts across various acute care settings. Therefore, there is a need to synthesize the literature on this topic now.

The objective of this scoping review was to comprehensively map and synthesize evidence on the enablers and barriers influencing NMs' understanding, use, and facilitation of EBP in acute care settings. A scoping review was deemed appropriate to identify these multilevel factors to inform the development of tailored interventions and support mechanisms.

## 2. Review Question

What enablers and barriers influence NMs' understanding, use, and facilitation of EBP in acute care settings?

### 2.1. Eligibility Criteria

#### 2.1.1. Population

This review focused on studies examining NMs, who are registered nurses in formal leadership roles responsible for overseeing nursing staff, coordinating care delivery, and managing clinical units, regardless of their educational background. Studies with mixed participant groups were excluded unless findings for NMs are reported separately.

#### 2.1.2. Concept

Studies must investigate at least one of the following aspects related to EBP: knowledge/understanding, attitudes, skills, leadership behaviors, implementation practices, influencing factors, or improvement interventions specifically for NMs. This included all types of studies using quantitative, qualitative, and mixed methods of primary research.

#### 2.1.3. Context

Focus was given to studies conducted in acute healthcare settings, such as hospitals, emergency departments, and intensive care units, where patients receive short-term, urgent, and specialized medical care. Studies conducted in outpatient, community, or other nonacute settings were excluded.

## 3. Method

The scoping review was conducted in accordance with the JBI methodology for scoping reviews and reported in accordance with PRISMA extension for scoping reviews (PRISMA-ScR) [29, 30]. The study protocol was registered on the Open Science Framework: https://osf.io/xbtym.

### 3.1. Search Strategy

The search strategy aimed to locate both published and unpublished studies. An initial limited search of MEDLINE and CINAHL was undertaken to identify articles on the topic. A comprehensive search was conducted across multiple electronic databases (MEDLINE, CINAHL, Cochrane Library, Scopus, JBI EBP database, Embase, Emcare, HMIC, and PsycINFO) using terms related to NMs, EBP, influencing factors, and acute hospitals (see Appendix 1). The reference list of all included sources of evidence was screened for additional studies, but none found. Only studies published in English were included regardless of publication date to be comprehensive.

### 3.2. Study Selection

Following the search, all identified citations were collated and uploaded to Rayyan (Rayyan & Co Ltd, Luton, UK) for deduplication and screening. Two reviewers independently (SS and LT) screened titles and abstracts and selected studies based on inclusion criteria. For all included studies, data on study characteristics, barriers, and enablers were extracted using a standardized form (Appendix 2). The extracted data were categorized according to the level of influence (personal and organizational) and type of factor (barrier and enabler).

**Figure 1:**
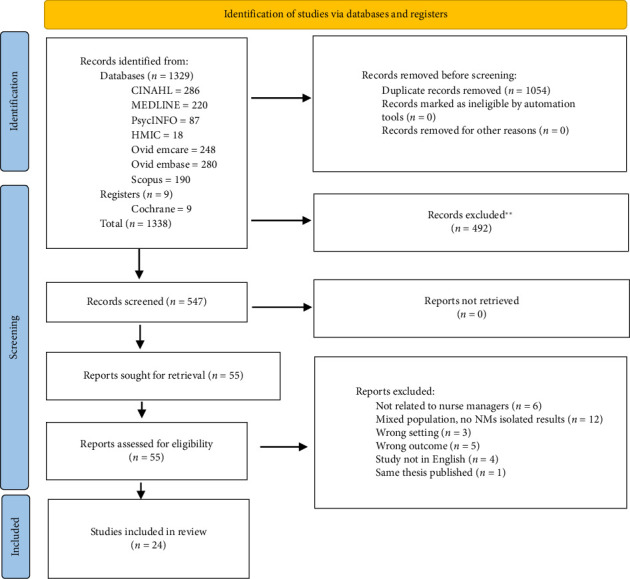
PRISMA flow diagram of search and selection of studies. ^∗∗^: All records were excluded by a human.

The full text of selected citations was assessed in detail against the eligibility criteria by two independent reviewers. Reasons for exclusion were papers that did not meet the eligibility criteria; these were recorded as presented in the PRISMA flowchart (Figure 1). Disagreements at each stage of the selection process were resolved through discussion.

### 3.3. Data Extraction

Data were extracted from papers included in the scoping review by one independent reviewer using a data extraction tool developed by the reviewers. Other reviewers did sample verification of extracted data. The data extracted included specific details about the participants, context, study methods, and key findings relevant to the review question. Extracted data are attached (Appendix 3). The studies were appraised with the Hawkers et al. [31] appraisal tool due to the heterogeneity of studies and for consistency of appraisal across studies.

### 3.4. Data Analysis and Presentation

The extracted data from included studies were analyzed using a mixed-methods approach. Quantitative data were analyzed using frequencies and counts. For textual data from qualitative studies, descriptive content analysis was employed to identify key themes and patterns. The results were presented in a combination of graphs, tables, and a narrative format to provide a comprehensive understanding of the enablers and barriers faced by NMs in implementing EBP.

The influencing factors were classified as enablers if they aid, support, or enhance the adoption of EBP and as barriers if they present challenges, obstacles, or limitations that impede, restrict, or prevent the successful implementation of EBP. These factors were also classified by source: personal factors, reflecting NMs' internal dispositions such as knowledge, skills, attitudes, and beliefs; and organizational factors, encompassing all external influences. Organizational factors were further differentiated into internal ones, present in the immediate work environment such as local policies and in-house training, and external ones, originating outside the setting, such as national policies, regulations, and external training programs.

## 4. Results

### 4.1. Study Characteristics

**Figure 2:**
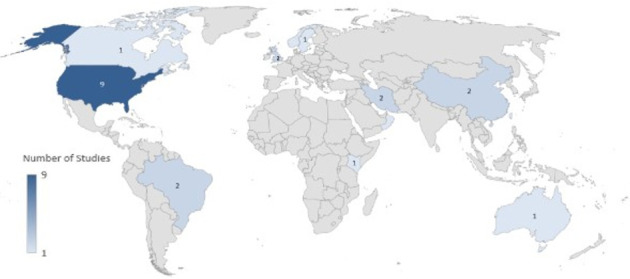
Geographical distribution of included studies.

**Figure 3:**
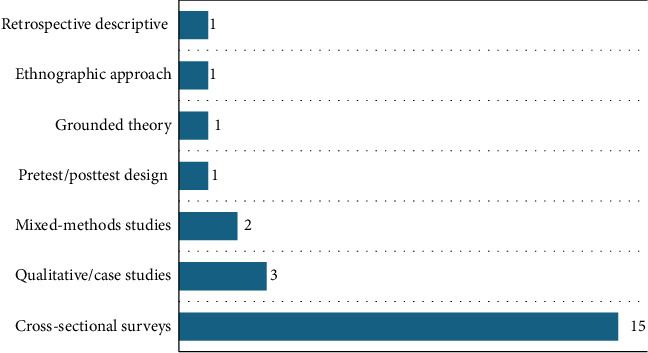
Distribution of methodologies used across the 24 studies.

This scoping review synthesized findings from 24 research studies conducted across 12 different countries: United States-9 [24, 32–39], the United Kingdom-2 [25, 32], Brazil-2 [40, 41], Iran-2 [42], China-2 [43, 44], Kenya-1 [45], Norway-1 [46], Oman-1 [47], Taiwan-1 [48], Australia-1 [49], Sweden-1 [50], and Canada-1 [51]. The distribution of studies across the world is presented in Figure 2. Studies reviewed spanned a range of methodologies, including cross-sectional surveys-15 [24, 34, 44], qualitative case studies-3 [25, 41], and mixed method studies-2 [36, 45] as shown in Figure 3, targeting NMs of varied levels in acute care settings [48] of varied sizes. Sample sizes range from qualitative studies with 18 participants to large-scale surveys involving over 1700 participants. These studies focused on understanding the roles of NMs in implementing EBP and assessing enablers and barriers to EBP use.

### 4.2. Methodological Rigor Included Studies

The Hawker et al. tool [31] was selected to appraise study quality due to its applicability across diverse research designs encountered in this scoping review. This tool addresses fundamental research quality elements while allowing flexible application to various methodologies. Each study was evaluated across nine domains using a four-point scale (1 = very poor to 4 = good): abstract clarity, introduction/aims, methodology, sampling, data analysis, ethics, results, transferability, and implications. The results of this appraisal are presented in Appendix 4.

All included reviews demonstrated robust quality (scoring > 27/36), with clear articulation of research questions, data sources, and practice implications. Notable limitations included inadequate ethics reporting in some studies (e.g., [51, 52]) and sampling constraints, with many employing convenience sampling or small samples that limited external validity. Quantitative studies typically utilized validated instruments (EBP Beliefs Scale, Implementation Leadership Scale, and BARRIERS questionnaire), while qualitative studies employed thematic analysis, grounded theory, or framework analysis.

Transferability was moderate across studies, with 68% of studies using single-site hospital data. Although data analysis was rigorous in most studies, some did not fully explain their analytical approaches, particularly regarding how themes were derived in qualitative studies. Several studies focused on specific contexts (magnet hospitals and teaching hospitals) with uncertain applicability to other settings (e.g., [36, 39]). The few multisite studies (e.g., [38, 43]) provided stronger external validity regarding organizational barriers to EBP adoption.

Despite these limitations, the overall high methodological quality provides a robust evidence base for understanding barriers and enablers to EBP among NMs in acute settings. However, future research should prioritize multisite or international comparative studies to enhance generalizability.

### 4.3. Enablers and Barriers to NMs' Understanding and Use of EBP

**Figure 4:**
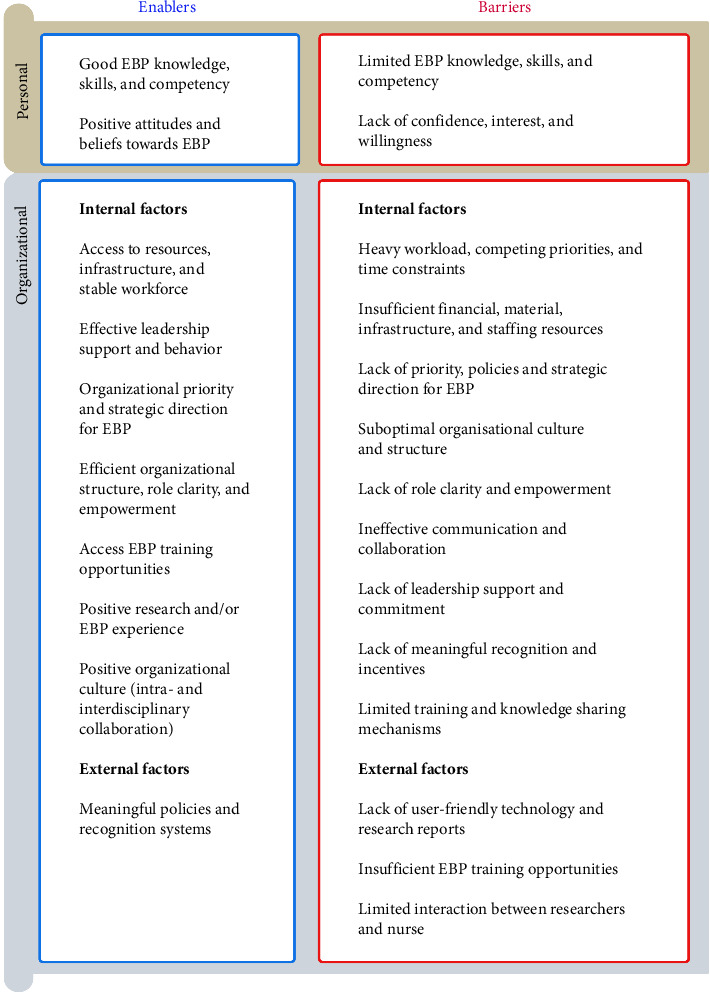
Enablers and barriers to NMs' understanding, use, and facilitation of EBP.

The synthesis of evidence from this scoping review reveals a complex interplay of factors influencing NMs' understanding, use, and facilitation of EBP as summarized in Figure 4.

### 4.4. EBP Enablers

#### 4.4.1. Personal Enablers

EBP implementation hinges significantly on NMs' personal capabilities and attitudes. Good EBP knowledge, skills, and competency were reported as essential to effectively implement EBP in many studies. This encompasses NMs' capacity to understand, interpret, and apply EBP as well as their ability to lead and promote EBP. NMs with strong research and critical appraisal skills are better equipped to identify, evaluate, and facilitate the application of relevant evidence [34, 37, 40, 43, 46, 50]. For example, Gallagher-Ford et al. [34] found a direct correlation between high EBP implementation and high EBP competency scores, which include skills in finding and appraising evidence. Camargo et al. [40] identified the ability to formulate a question and review practice as essential for identifying and applying relevant evidence. Additionally, increased ability to use internet-based resources and access to libraries and electronic databases has been linked to improved access to research evidence [48, 51].

Furthermore, positive attitudes and beliefs towards EBP were also identified as significant personal enablers. This involves the NMs' disposition of confidence in understanding, leading, and applying EBP, alongside recognizing its value in improving outcomes and decision-making, and a commitment to professional growth. Thus, NMs with such positive attitudes and beliefs are more likely to integrate it into their practice, contributing to the overall success of EBP initiatives [33, 34, 38, 41–43, 47, 48, 50].

#### 4.4.2. Organizational Enablers (Internal Factors)

Numerous internal organizational factors contribute to the successful implementation of EBP. Key among these is access to resources, infrastructure, and a stable workforce as emphasized in multiple studies [36, 39, 41, 43, 46, 49, 53]. This enabler refers to the availability of sufficient material, financial, and human resources necessary to implement and sustain EBP initiatives. A well-established infrastructure, such as libraries and electronic bibliographic databases, and dedicated time for EBP activities support evidence retrieval and application [41, 51, 53]. The stability of the workforce and availability of resources, in conjunction with strong leadership support, create a favorable environment for EBP integration and sustainability. Ensuring that staff have the tools and resources was consistently associated with successful EBP implementation.

Effective leadership support and behavior also emerged as critical enablers across multiple studies [24, 25, 32, 33, 35–37, 39, 46, 49, 53]. This entails leaders who actively champion EBP, provide visible support, and model evidence-based decision-making. Gallagher-Ford et al. [34] identified senior leadership support as significantly promoting EBP implementation within the United States. Shuman et al. [24] confirmed that proactive, knowledgeable, and supportive NMs fostered a positive climate for EBP. Additionally, Renolen et al. [46] and Patton et al. [37] emphasized that when NMs actively inspired and facilitated teamwork, EBP engagement increased. Furthermore, the presence of dedicated EBP champions, such as nurse directors and NMs, was noted to be crucial in fostering a culture conducive to EBP in the UK [25].

Another factor influencing EBP success is the priority and strategic direction for EBP within an organization. This is the explicit positioning of EBP as a core organizational value and strategic imperative. It includes formal recognition of EBP in strategic plans and operational priorities, alongside setting expectations, providing a structured environment for EBP, and integrating it into the core functions of the organization [34–36, 46, 50]. Organizations that emphasize the value and importance of EBP help to create a culture that prioritizes and consistently communicates EBP goals [33, 35]. In such settings, clear strategic direction allows for focused implementation.

Efficient organizational structure, role clarity, and empowerment also play a significant role. This refers to organizational designs with clear delineation of responsibilities and appropriate delegation of authority to enact evidence-based changes. Empowerment of nurses, including both leaders and frontline staff, through clear communication, appropriate task delegation, and involvement in decision-making processes were highlighted as crucial [25, 32, 35, 45, 46, 53]. For instance, Wilkinson et al. [25] noted that empowering nurses with clear roles and communication channels facilitated EBP implementation. Similarly, Kitson et al. [49] found that selecting focused areas for EBP implementation, aligned with nursing strengths, promoted better outcomes. When roles are well-defined and staff are empowered to participate in decision-making, the implementation process becomes more streamlined and effective [34, 36, 37, 41, 43, 46, 50, 52].

Access to EBP training opportunities further enhances EBP implementation. This involves the organizational facilitation of formal and informal learning opportunities that develop staff capability including leadership, evidence appraisal, implementation, and evaluation. Workshops and structured training, especially when tailored to specific needs, were the most successful strategies [40, 49]. Camargo et al. [40] and Chen et al. [43] stressed the importance of real-world problem discussions, peer interactions, and effective EBP education as key enablers. Camargo et al. [40] found that workshops using a hermeneutic-dialectic approach were particularly effective in enhancing EBP understanding among NMs in Brazil. These interactive workshops, addressing practical challenges, not only provided critical knowledge but also facilitated discussions on practical challenges, leading to improved EBP uptake. These findings suggest that hands-on, participatory approaches are more effective in engaging NMs and fostering a deeper understanding of EBP principles.

Likewise, positive research and/or EBP experience, which relates to a NMs' previous successful encounters with aspects of research and EBP activities such as conducting research, utilizing research findings, or implementing EBP, supports EBP success. Such experience, whether gained through formal education, structured training, or practice-based engagement, enhances NMs' confidence, critical appraisal skills, and a more proactive approach to EBP [24, 34, 40, 46, 50, 51]. For example, Johansson et al. [50] and Gallagher-Ford et al. [34] linked higher education to increased EBP engagement, but this was attributed to the experiential aspects of that education, such as hands-on research training and exposure to evidence-based decision-making. Similarly, Renolen et al. [46] and Camargo et al. [40] showed that managers who had participated in practical workshops or prior EBP activities were more proactive and effective in supporting EBP in practice. Therefore, it is not knowledge in isolation, but positive, practical experience that most strongly supports EBP implementation. Accordingly, Gallagher-Ford et al. [34] specifically found that nurses with higher degrees had increased implementation rates, likely due to their positive formal training in research methods and evidence-based decision-making. Although holding a higher degree does not automatically guarantee a positive research experience, higher degrees typically have increased exposure to structured research education, advanced critical appraisal skills, and greater familiarity with evidence utilization, which collectively enhance their capability and willingness to implement EBP [34, 50]. Additionally, participating in aspects of research such as recruitment in trials, having access to research committees, and academic databases, as Royle et al. [51] noted, further contributed to positive outcomes.

A positive organizational culture which encompasses collaborative relationships, shared values supporting EBP, and intra- and interdisciplinary cooperation that facilitates knowledge exchange and implementation of best significantly enhances EBP engagement. Six studies [25, 32, 35, 43, 46, 49] have identified such collaboration as a crucial enabler of EBP success. Shuman et al. [24] emphasized that collaboration fosters a positive climate for EBP, while Kitson et al. [49] and Johansson et al. [50] found that teamwork across professional boundaries enhances success. Additionally, external partnerships with academic institutions and healthcare organizations provide valuable resources and expertise, further supporting EBP efforts [32, 39, 49, 50].

#### 4.4.3. Organizational Enablers (External Factors)

Meaningful policies and recognition systems are the sole external factor identified as an enabler in three studies [34, 35, 39]. This refers to fair, transparent, and inclusive policies and practices that prioritize staff and patient well-being, coupled with unbiased recognition systems designed to enhance both morale and performance across the workforce and organization. Meaningful policies and recognition systems highlight the importance of external validation and quality assurance programs in promoting EBP. These external organizational enablers were associated with a culture that values EBP, promotes continuous improvement, and provides the necessary resources for sustained implementation [34–36, 39, 45, 48].

### 4.5. EBP Barriers

#### 4.5.1. Personal Barriers

At the personal level, the most significant barrier appears to be limited EBP knowledge, skills, and competency among NMs. This occurs when NMs lack sufficient understanding and practical ability to interpret, implement, and lead EBP. This was a recurring theme across many studies, including Gallagher-Ford et al. [34], Camargo et al. [40], and Lai et al. [44]. Low confidence and lack of EBP skills hindered implementation efforts [34, 42]. Additionally, Camargo et al. [40] reported that nurses often struggled with understanding research articles and lacked knowledge on how to search for evidence, further obstructing EBP adoption. This difficulty in understanding and applying research underscores the need for targeted educational interventions. The knowledge and competency barrier are closely tied to organizational factors, as it relates to limited training and knowledge-sharing mechanisms within healthcare institutions.

The lack of confidence, interest, and willingness were also a reported personal barrier. This refers to a NMs' absence of enthusiasm, self-confidence, or curiosity in understanding or applying EBP [34, 42, 44, 49, 53].

#### 4.5.2. Organizational Barriers (Internal)

Organizational barriers, particularly internal factors, appear to be the most prevalent and multifaceted. The most frequently cited organizational barrier across studies is the combination of high workload, competing priorities, and time constraints. This issue, where excessive operational demands and conflicting responsibilities limit the time and capacity available to engage in EBP, was highlighted by numerous researchers as pervasive, including Gallagher-Ford et al. [34], Wilkinson et al. [25], and Camargo et al. [40], among others. Shuman et al. [24] highlighted that in the United States, NMs often faced overloaded roles and competing demands, which left little time for engaging with EBP activities. This was echoed by Wilkinson et al. [25] in the United Kingdom, where the heavy workloads of NMs were seen as a major impediment to EBP implementation. Similarly, Kitson et al. [49] noted that time pressures and heavy workloads negatively impact EBP activities. The consistency of this finding across studies spanning from 1997 to 2024 suggests that time pressure remains a persistent challenge in the nursing field.

Insufficient financial, material, infrastructure, and staffing resources also present significant barriers in many studies including Camargo et al. [41], Lai et al. [44], and Barako et al. [45]. This resource scarcity is closely related to and manifested as heavy workload and time constraints as experienced by understaffed teams. Camargo et al. [41] and Renolen et al. [46] reported that limited financial support and a lack of infrastructure can demotivate staff and make EBP implementation challenging. Additionally, a lack of priority, policies, and strategic direction for EBP within an organization was cited as a major hindrance by Lai et al. [44] and Shuman et al. [38], with both studies indicating that a lack of focus on EBP at the strategic level detracted from progress.

Another challenge is suboptimal organizational culture and structure. Kitson et al. [49] noted that resistance to change and siloed work practices impeded collaboration, while Weng et al. [48] found that cultures focused on task completion left little room for EBP activities. Studies by Gallagher-Ford et al. [34], Shuman et al. [24], and Renolen et al. [46] have also highlighted how suboptimal organizational cultures can impede EBP implementation. Additionally, variability in organizational climates was cited as a barrier. Shuman et al. [24] reported that this variability led to inconsistencies in EBP implementation across different units, further complicating efforts to standardize practices. A lack of role clarity and empowerment was similarly problematic, with Wilkinson et al. [25] and Camargo et al. [41] pointing to unclear roles and hierarchical structures that hindered nurses' involvement in EBP. Ineffective communication and collaboration further exacerbate EBP barriers. Lai et al. [44] and Kitson et al. [49] highlighted poor communication and lack of collaboration as major obstacles.

Lack of leadership support and commitment also remains a key issue, with Kueny et al. [35] and Shuman et al. [38] emphasizing that insufficient leadership backing diminishes the impact of EBP efforts. Wilkinson et al. [25], Kueny et al. [35], and Patton et al. [37] all emphasized how a lack of support from higher management can significantly hinder EBP implementation. This is further compounded by a lack of role clarity and empowerment, as noted by Wilkinson et al. [25] and Camargo et al. [41].

Moreover, lack of meaningful recognition and incentives weakens motivation for EBP implementation. Shuman et al. [38] observed that EBP efforts often go unrewarded, limiting enthusiasm. Finally, limited training and knowledge-sharing mechanisms present barriers, as noted by Camargo et al. [40] and Hasanpoor et al. [53], who reported that insufficient training and difficulty in accessing research knowledge inhibited EBP integration.

#### 4.5.3. Organizational Barriers (External Factors)

External organizational barriers include a lack of user-friendly technology, technically complex worded research papers, and insufficient EBP training opportunities. Studies by Royle et al. [51] and Hasanpoor et al. [53] have identified limited access to electronic journals and insufficient training in using electronic databases and conducting effective searches as significant challenges. Additionally, research by Lai et al. [44], Royle et al. [51], and Almaskari [47] underscores the lack of EBP training opportunities for NMs as a critical barrier. Furthermore, limited interaction between researchers and clinical nurses often impedes the integration of EBP, as described by Hasanpoor et al. [53] and Camargo et al. [41]. It is important to note that studies from nearly 4 decades ago [51, 52] highlighted the issue of nonuser-friendly technology with cumbersome interfaces, limited search functionality, and restrictive data handling options. However, given significant technological advancements and improvements in electronic journal access and research paper reporting since then, it is possible that managers' current experiences may differ. Nonetheless, there remains a need for more user-friendly technology to ease finding research resources and clearer presentation of findings to ensure effective utilization. Although these external organizational factors are mentioned less frequently, they do impact EBP implementation.

### 4.6. EBP Interventions for NMs in Acute Care

Several interventions in seven studies across high- and upper-middle-income countries have aimed to strengthen EBP capabilities among NMs in acute care settings, with varied degrees of success (Appendix 5). Short-term improvements in EBP knowledge, confidence, and engagement were commonly reported following leadership workshops [40], reflective dialog-based interventions [41], and formal education programs [50]. National-scale initiatives like Taiwan's TEBMA program demonstrated reductions in perceived barriers to EBP [48], while structured organizational efforts in magnet hospitals and through KT toolkits enhanced staff attitudes and team cohesion [39, 49]. However, the translation of these gains into observable, sustained practice change remains limited. Notably, some interventions, such as the leadership behavior program by Patton et al. [37], revealed a disconnect between NMs' self-reported improvements and the perceptions of their clinical staff, raising questions about the visibility and impact of leadership behavior change.

The findings underscore the critical, yet complex, relationship between EBP training, organizational context, and leadership behavior. While many interventions improved individual-level knowledge and attitudes, few translated into long-term practice change or observable improvements in managerial leadership. The absence of longitudinal data and reliance on self-report measures limit claims of sustained impact. Moreover, the observed discrepancy between perceived and actual behavior change suggests that knowledge acquisition alone is insufficient, requiring deeper cultural and structural shifts within organizations for effective implementation of EBP.

## 5. Discussion

The findings of this scoping review are discussed through the lens of Bandura's reciprocal determinism theory [54]. The theory posits that human functioning is shaped by a continuous and dynamic interaction between three key elements: personal factors (such as beliefs, attitudes, and cognitive abilities), behavior (actions and choices made by an individual), and the environment (social and organizational influences). Rather than viewing these elements as separate or independent forces, reciprocal determinism emphasizes their mutual influence, meaning that a person's thoughts and beliefs shape their actions, which in turn influence and are influenced by their environment. This theoretical framework provides a lens through which to understand the complex interactions observed between personal factors, leadership behaviors, and organizational environment in the context of EBP implementation among NMs.

A central finding of this review is the primacy of personal factors in influencing EBP engagement and facilitation among NMs. These factors consistently emerged as the most decisive determinants of successful EBP implementation, even when organizational barriers are similar [35, 40, 46]. Specifically, positive attitudes, strong self-efficacy, and foundational EBP and leadership knowledge and skills are consistently associated with greater uptake [34, 40, 42, 46, 48].

These personal factors are not static; rather, they dynamically influence and reinforce each other. Positive attitudes stimulate competency development, while growing competencies further strengthens confidence and commitment towards EBP. This reciprocal reinforcement empowers NMs to navigate and overcome persistent barriers such as time constraints, resource limitations, and lack of authority [25, 44, 46]. This explains why NMs with strong personal factors can overcome seemingly insurmountable barriers, while a lack of belief or commitment can undermine efforts despite resource availability [37, 44]. These findings reinforce the need for interventions that not only deliver knowledge but also engage with the critical attitudinal and behavioral dimensions of EBP leadership [55–60].

This review found that personal barriers related to attitudes, beliefs, and knowledge persist across all income levels, although in context-specific forms. In high-income countries, these barriers often stem from cultural resistance to change, practice inertia, or perceived disconnects between research evidence and clinical realities. Many NMs in these settings report uncertainty in interpreting research or discomfort challenging traditional norms despite access to high-quality databases and formal training. In low- and middle-income countries (LMICs), personal barriers are frequently more foundational, rooted in limited exposure to EBP during training, inadequate research access, and low baseline awareness of EBP principles.

These differences highlight that while influencing factors are consistent in type, they differ in manifestation and impact, shaped by systemic, educational, and cultural contexts. Across all income settings, the recurring barrier of limited self-efficacy undermines NM' motivation to engage with EBP even when resources are available [34, 42, 44, 49, 53]. Bandura's emphasis on self-efficacy as a key determinant of behavior is clearly supported in this review, identifying it as a critical, modifiable factor in enhancing EBP implementation.

Leadership behaviors of NMs, representing the “behavior” component in Bandura's model, emerged as crucial for EBP facilitation. Proactive, knowledgeable, supportive, and perseverant NMs effectively model EBP and actively facilitate EBP. They champion EBP among staff, recognize and reward EBP efforts, allocate protected time for EBP activities, and foster psychologically safe environments that encourage enquiry and innovation [24, 32, 34, 35, 38, 44, 46, 49, 53]. These behaviors significantly contribute to a positive organizational environment conducive to EBP, even in the face of initial barriers.

These EBP-enabling behaviors largely align with transformational leadership, with some elements of servant leadership also evident [61–64]. This consistency with wider research demonstrates transformational leadership's positive association with nurses' use of EBP, job satisfaction, empowerment, and innovation, all of which support successful EBP integration into clinical practice [61, 62, 65]. Furthermore, interventions that improve leadership behaviors have also been shown to enhance EBP competencies, confirming a reciprocal influence between behavior and personal attributes [37]. Consequently, NMs must cultivate these leadership capabilities to fuel leadership behaviors that will shape supportive EBP environments [24]. This is congruent with the broader EBP literature which underscore the crucial role of facilitation in EBP adoption [37, 46, 66–68]. Leadership that champions EBP acts as a catalyst, empowering staff, normalizing evidence use, and reinforcing self-efficacy at both individual and team levels. Conversely, passive or unsupportive leadership behaviors are associated with weaker EBP cultures and greater implementation barriers.

Organizational factors, aligning with Bandura's “environment” component, are crucial for supporting EBP. A supportive organizational environment features strategic leadership commitment, robust resource allocation, and institutional structures that embed EBP as a core value [35, 44, 48]. Enabling conditions include clear role definitions [25, 46], participatory decision-making and interdisciplinary collaboration [46, 49], and access to targeted professional development opportunities, such as interactive, problem-focused workshops [37, 40]. These structures enhance NMs' capability and confidence in leading evidence-informed changes.

However, limited time remains a universal organizational barrier, irrespective of economic context. Whether arising from competing priorities in high-income countries or severe understaffing in LMICs, overwhelming clinical responsibilities consistently impede NMs' capacity to engage with EBP [34, 41, 53]. This highlights a fundamental lapse in healthcare systems' role design and strategic planning, where EBP-related responsibilities are often deprioritized or insufficiently resourced [25, 44]. Addressing this systemic issue requires restructuring healthcare systems and redefining job roles to realistically incorporate the operationalization of EBP as a core responsibility.

Organizational support also shows clear economic variation. In high-income countries, resources such as digital libraries, protected time policies, and EBP leadership roles are generally systematic and sustainable [24, 34]. Yet, uneven EBP implementation remains, often reflecting fragmented leadership or lack of integration into routine workflows. In contrast, middle- and lower income settings typically rely on short-term, externally driven efforts, such as international collaborations, grant-funded workshops, or isolated institutional initiatives [40, 53]. While impactful, these interventions are rarely sustainable, hinder systemic integration, and reinforce the vulnerability of EBP initiatives in these settings. This underscores the need for adequate healthcare resourcing, local capacity-building, and institutional ownership.

In conclusion, the findings of this review emphasize that personal factors are critical for generating the evidence component in the EBP process to inform nursing practice. EBP competency is strongly associated with higher EBP implementation rates [34, 40, 48, 51]. Leadership behaviors, meanwhile, significantly contribute to cultivating organizational environments that sustain EBP [24, 34]. The interaction between these elements (personal, behavioral, and environmental) completes the reciprocal cycle proposed by Bandura. This dynamic is exemplified by the work of Warren et al. [39], who found that multifaceted interventions implemented to achieve and maintain magnet designation led to a positive shift in clinical NMs' attitudes towards EBP. It highlights how personal capabilities, leadership behaviors, and access to resources intertwine to enable nurses to find and apply evidence in their practice. Given this interdependence, the need for ongoing focused education and training in EBP as frequently observed across the included studies is both appropriate and justified to strengthen both personal factors and behaviors related to EBP. Framing these findings within Bandura's reciprocal determinism theory offers a robust theoretical foundation for future research and interventions aimed at enhancing NMs' engagement with EBP.

### 5.1. Findings Compared to Wider Evidence

Although the scoping review focuses on NMs, several of its findings resonate across the wider nursing and healthcare workforce. While lack of time, limited self-efficacy, and conceptual ambiguity around EBP affect various healthcare professionals [69–73], their implications differ by role. Clinical nurses with limited EBP skills may struggle individually, whereas managers with similar deficits risk perpetuating outdated practices system-wide through resource and policy decisions. Consequently, although EBP competencies are vital at all levels, their transformational potential is most pronounced in leadership roles. NMs are uniquely positioned to foster enabling conditions for EBP, requiring a distinct skillset that blends EBP proficiency with leadership, management, and political acumen to navigate organizational dynamics, secure resources for evidence implementation, and align EBP initiatives with institutional strategic goals [74–78].

### 5.2. Strengths and Limitations

To our knowledge, this is the first scoping review to explore enablers and barriers EBP among NMs in acute care settings. By applying Bandura's reciprocal determinism theory, we offer a unique perspective on the intricate interplay of factors influencing EBP use in this context. Another strength is the high methodological quality of included studies. However, this scoping review is limited by its English-only search, and the potential omission of studies from nonindexed journals.

## 6. Conclusions

This scoping review provides a comprehensive overview of the enablers and barriers to EBP among NMs in acute care settings. Key findings highlight the primacy of personal factors, including positive attitudes, beliefs, and strong EBP competencies, as well as supportive leadership behaviors in fostering a positive organizational environment for EBP. The study also emphasizes the need for adequate resources, infrastructure, clear communication, and strategic direction to enable EBP implementation. These findings underscore the complex relationship of factors influencing EBP implementation and offer valuable insights for healthcare organizations seeking to improve EBP integration.

### 6.1. Implications for Nursing Management and Practice

The findings recognized NMs in acute care settings as key agents in advancing EBP. Beyond knowledge dissemination, successful EBP implementation requires a deliberate focus on developing the personal dispositions and leadership behaviors that support evidence-informed decisions. As such, healthcare organizations must (1) invest in comprehensive EBP leadership development combining technical training with behavioral transformation through targeted training, mentorship, peer learning, and practical application opportunities; (2) formally embed EBP leadership in NMs' job descriptions, performance metrics, and workload allocations with protected time for implementation activities; and (3) establish organizational structures that prioritize EBP through clearly defined roles, clear academic-clinical communication channels, equitable resource access, and meaningful recognition systems that reward evidence-based innovations while fostering psychological safety.

### 6.2. Implications for Future Research

Given the gaps found, the following are recommended for further research: (1) Develop and test interventions that specifically aim to shape NMs' attitudes, beliefs, and leadership behaviors alongside traditional knowledge-based training. These intervention studies are encouraged to use validated instruments to assess changes in self-efficacy, leadership styles, and EBP engagement and consistently and transparently reporting for comparative analysis across studies and better assessment of intervention effectiveness. (2) Adopt longitudinal designs that evaluate whether initial improvements in EBP knowledge and attitudes are sustained and translated into long-term behavioral and organizational change. Follow-up periods of 3–5 years would provide insights into the durability of interventions and the conditions that support or hinder sustained implementation. (3) Multicomponent interventions that simultaneously address all personal, behavioral, and environmental domains should be developed and evaluated given their interdependence. These strategies can be compared to single-focus interventions to determine relative effectiveness. Mixed method designs and objective outcome measures, such as audit data, staff engagement metrics, and patient outcomes, should be prioritized over sole reliance on self-report tools, ensuring a more accurate picture of impact and sustainability.

## Data Availability

The data that support the findings of this study are available in the supporting information of this article.

## Supporting Information

Supporting Information 1 Appendix 1: Search strategies for the review.docx.

Supporting Information 2 Appendix 2: Data extraction instrument.docx.

Supporting Information 3 Appendix 3: Extracted data for the study.docx.

Supporting Information 4 Appendix 4: Quality appraisal of included studies.

Supporting Information 5 Appendix 5: EBP Interventions for nurse mangers in acute care.
