# Supplementary material for: Understanding, Using, and Facilitating Evidence-Based Practice: A Scoping Review of Influencing Factors Among Nurse Managers in Acute Care
Source: J Nurs Manag. 2025 Jul 4;2025:2155376. doi: 10.1155/jonm/2155376 (PMC12253997; doi:10.1155/jonm/2155376)
Supplement: Supporting Information 1 — Appendix 1: Search strategies for the review.docx. [file 2155376.f1.docx]

### Appendix 1: Search strategies for the review

**CINAHL (EBSCOhost): Search updated on April 18, 2024.**

| **Search** | **Query** | **Records retrieved** |
| --- | --- | --- |
| S1 | TI ( "Nurs* manager*" OR "Nurs* administrator*" OR "Nurs* leader*" OR "Charge Nurse*" OR "Ward Manager*" OR "Unit Manager*" OR "Nurs* supervisor*" ) OR AB ( "Nurs* manager*" OR "Nurs* administrator*" OR "Nurs* leader*" OR "Charge Nurse*" OR "Ward Manager*" OR "Unit Manager*" OR "Nurs* supervisor*" ) | 17,676 |
| S2 | (MM "Nursing Leaders") OR (MM "Clinical Nurse Leaders") OR (MM "Nurse Managers+") OR (MM "Charge Nurses") OR (MM "Nurse Administrators") | 19,167 |
| S3 | S1 OR S2 | 31,368 |
| S4 | TI ( "Evidence-based pract*" OR "Evidence-Informed pract*" OR "Evidence-based healthcare" OR "Evidence-based Nursing" OR "EBP" OR "EBN" OR "Evidence implementation" OR "research utili?ation" ) OR AB ( "Evidence-based pract*" OR "Evidence-Informed pract*" OR "Evidence-based healthcare" OR "Evidence-based Nursing" OR "EBP" OR "EBN" OR "Evidence implementation" OR "research utili?ation" ) | 19,785 |
| S5 | (MM "Nursing Practice, Evidence-Based") OR (MM "Nursing Practice, Research-Based") OR (MM "Nursing Practice, Theory-Based") | 12,056 |
| S6 | S4 OR S5 | 27,843 |
| S7 | TI ( Factor* OR Influence* OR Facilitat* OR Determinant* OR Barrier* OR Enabler* OR Knowledge OR Skill* OR Attitude* OR Perception* OR Belief* OR "Learning need*" OR Practice OR Support* OR Promote* OR Competenc* ) OR AB ( Factor* OR Influence* OR Facilitat* OR Determinant* OR Barrier* OR Enabler* OR Knowledge OR Skill* OR Attitude* OR Perception* OR Belief* OR "Learning need*" OR Practice OR Support* OR Promote* OR Competenc* ) | 2,309,878 |
| S8 | (MM "Nursing Knowledge") OR (MM "Knowledge") OR (MM "Nurse Attitudes") OR (MM "Midwife Attitudes") OR (MM "Attitude to Change") OR (MM "Nursing Practice") OR (MM "Decision Support Systems, Clinical") OR (MM "Training Support, Financial") OR (MM "Work Environment") OR (MM "Research Support") OR (MM "Information Needs") OR (MM "Clinical Competence") OR (MM "Nursing Skills") OR (MM "Power") OR (MM "Politics") OR (MH "Leadership") OR (MM "Teaching Materials") OR (MM "Reference Tools") OR (MM "Information Resources") OR (MM "Nursing Labor Supply") | 216,850 |
| S9 | S7 OR S8 | 2,429,137 |
| S10 | TI ( "Acute care" OR Hospital* OR "Inpatient setting*" ) OR AB ( "Acute care" OR Hospital* OR "Inpatient setting*" ) | 570,300 |
| S11 | (MM "Acute Care") OR (MM "Inpatients") OR (MM "Infant, Hospitalized") OR (MM "Hospitalization of Older Persons") OR (MM "Adolescent, Hospitalized") OR (MM "Child, Hospitalized") | 22,588 |
| S12 | S10 OR S11 | 576,575 |
| S13 | S3 AND S6 AND S9 AND S12 | 286 |

**MEDLINE (EBSCOhost): Search updated on May 15, 2024.**

| **Search** | **Query** | **Records retrieved** |
| --- | --- | --- |
| S1 | TI ( "Nurs* manager*" OR "Nurs* administrator*" OR "Nurs* leader*" OR "Charge Nurse*" OR "Ward Manager*" OR "Unit Manager*" OR "Nurs* supervisor*" ) OR AB ( "Nurs* manager*" OR "Nurs* administrator*" OR "Nurs* leader*" OR "Charge Nurse*" OR "Ward Manager*" OR "Unit Manager*" OR "Nurs* supervisor*" ) | 13,878 |
| S2 | (MM "Nurse Administrators") | 10,899 |
| S3 | S1 OR S2 | 21,406 |
| S4 | TI ( "Evidence-based pract*" OR "Evidence-Informed pract*" OR "Evidence-based healthcare" OR "Evidence-based Nursing" OR "EBP" OR "EBN" OR "Evidence implementation" OR "research utili?ation" ) OR AB ( "Evidence-based pract*" OR "Evidence-Informed pract*" OR "Evidence-based healthcare" OR "Evidence-based Nursing" OR "EBP" OR "EBN" OR "Evidence implementation" OR "research utili?ation" ) | 26,114 |
| S5 | (MM "Evidence-Based Nursing") OR (MM "Evidence-Based Practice") | 8,162 |
| S6 | S4 OR S5 | 30,913 |
| S7 | TI ( Factor* OR Influence* OR Facilitat* OR Determinant* OR Barrier* OR Enabler* OR Knowledge OR Skill* OR Attitude* OR Perception* OR Belief* OR "Learning need*" OR Practice OR Support* OR Promote* OR Competenc* ) OR AB ( Factor* OR Influence* OR Facilitat* OR Determinant* OR Barrier* OR Enabler* OR Knowledge OR Skill* OR Attitude* OR Perception* OR Belief* OR "Learning need*" OR Practice OR Support* OR Promote* OR Competenc* ) | 10,436,458 |
| S8 | TI ( "Acute care" OR Hospital* OR "Inpatient setting*" OR "Critical care" OR "Emergency care" OR "Intensive Care") OR AB ( "Acute care" OR Hospital* OR "Inpatient setting*" ) OR AB ( "Acute care" OR Hospital* OR "Inpatient setting*" OR "Critical care" OR "Emergency care" OR "Intensive Care") | 1,848,877 |
| S9 | (MM "Inpatients") OR (MM "Hospitalization") OR (MM "Hospitals") OR (MM "Adolescent, Hospitalized") OR (MM "Child, Hospitalized") OR (MM "Critical Care") OR (MM "Critical Care Nursing") OR (MM "Acute Care Surgery") OR (MM "Hospitals") | 153,767 |
| S10 | S8 OR S9 | 1,888,304 |
| S11 | S3 AND S6 AND S7 AND S10 | 220 |

**APA PsycINFO <1806 to May Week 2 2024>**

| **Search** | **Query** | **Records retrieved** |
| --- | --- | --- |
| 1 | ("Nurs* manager*" or "Nurs* administrator*" or "Nurs* leader*" or "Charge Nurse*" or "Ward Manager*" or "Unit Manager*" or "Nurs* supervisor*").ab. or ("Nurs* manager*" or "Nurs* administrator*" or "Nurs* leader*" or "Charge Nurse*" or "Ward Manager*" or "Unit Manager*" or "Nurs* supervisor*").ti. | 4125 |
| 2 | exp Evidence Based Practice/ | 21685 |
| 3 | ("Evidence-based pract*" or "Evidence-Informed pract*" or "Evidence-based healthcare" or "Evidence-based Nursing" or EBP or EBN or "Evidence implementation" or "research utili$ation").ab. or ("Evidence-based pract*" or "Evidence-Informed pract*" or "Evidence-based healthcare" or "Evidence-based Nursing" or EBP or EBN or "Evidence implementation" or "research utili$ation").ti. | 13170 |
| 4 | (Factor* or Influence* or Facilitat* or Determinant* or Barrier* or Enabler* or Knowledge or Skill* or Attitude* or Perception* or Belief* or "Learning need*" or Practice or Support* or Promote* or Competenc*).ab. or (Factor* or Influence* or Facilitat* or Determinant* or Barrier* or Enabler* or Knowledge or Skill* or Attitude* or Perception* or Belief* or "Learning need*" or Practice or Support* or Promote* or Competenc*).ti. | 2888841 |
| 5 | exp Intensive Care/ or exp Hospitals/ or *Emergency Services/ | 44707 |
| 6 | ("Acute care" or Hospital* or "Inpatient setting*" or "Critical care" or "Emergency care" or "Intensive Care").ab. or ("Acute care" or Hospital* or "Inpatient setting*" or "Critical care" or "Emergency care" or "Intensive Care").ti. | 195434 |
| 7 | 5 or 6 | 205491 |
| 8 | 2 or 3 | 27587 |
| 9 | 1 and 7 and 8 | 87 |

**HMIC Health Management Information Consortium <1979 to March 2024>**

| **Search** | **Query** | **Records retrieved** |
| --- | --- | --- |
| 1 | exp nurse managers/ | 875 |
| 2 | ("Nurs* manager*" or "Nurs* administrator*" or "Nurs* leader*" or "Charge Nurse*" or "Ward Manager*" or "Unit Manager*" or "Nurs* supervisor*").mp. [mp=title, other title, abstract, heading words] | 2049 |
| 3 | 1 or 2 | 2346 |
| 4 | exp Evidence based practice/ | 2620 |
| 5 | ("Evidence-based pract*" or "Evidence-Informed pract*" or "Evidence-based healthcare" or "Evidence-based Nursing" or EBP or EBN or "Evidence implementation" or "research utili$ation").mp. [mp=title, other title, abstract, heading words] | 3173 |
| 6 | 4 or 5 | 3173 |
| 7 | hospitals/ or exp Acute services/ or exp Acute care/ | 10748 |
| 8 | ("Acute care" or Hospital* or "Inpatient setting*" or "Critical care" or "Emergency care" or "Intensive Care").mp. [mp=title, other title, abstract, heading words] | 61241 |
| 9 | 7 or 8 | 61558 |
| 10 | 3 and 6 and 9 | 18 |

**Ovid Emcare <1995 to 2024 Week 19>**

| **Search** | **Query** | **Records retrieved** |
| --- | --- | --- |
| 1 | exp nurse manager/ or exp nurse administrator/ | 8773 |
| 2 | ("Nurs* manager*" or "Nurs* administrator*" or "Nurs* leader*" or "Charge Nurse*" or "Ward Manager*" or "Unit Manager*" or "Nurs* supervisor*").ti. or ("Nurs* manager*" or "Nurs* administrator*" or "Nurs* leader*" or "Charge Nurse*" or "Ward Manager*" or "Unit Manager*" or "Nurs* supervisor*").ab. | 11695 |
| 3 | 1 or 2 | 15418 |
| 4 | *evidence based practice/ or exp evidence based nursing/ | 13813 |
| 5 | ("Evidence-based pract*" or "Evidence-Informed pract*" or "Evidence-based healthcare" or "Evidence-based Nursing" or EBP or EBN or "Evidence implementation" or "research utili$ation").ti. or ("Evidence-based pract*" or "Evidence-Informed pract*" or "Evidence-based healthcare" or "Evidence-based Nursing" or EBP or EBN or "Evidence implementation" or "research utili$ation").ab. | 17495 |
| 6 | 4 or 5 | 26853 |
| 7 | (Factor* or Influence* or Facilitat* or Determinant* or Barrier* or Enabler* or Knowledge or Skill* or Attitude* or Perception* or Belief* or "Learning need*" or Practice or Support* or Promote* or Competenc*).ti. or (Factor* or Influence* or Facilitat* or Determinant* or Barrier* or Enabler* or Knowledge or Skill* or Attitude* or Perception* or Belief* or "Learning need*" or Practice or Support* or Promote* or Competenc*).ab. | 3212049 |
| 8 | hospitals/ or exp Acute services/ or exp Acute care/ | 152469 |
| 9 | ("Acute care" or Hospital* or "Inpatient setting*" or "Critical care" or "Emergency care" or "Intensive Care").ti. or ("Acute care" or Hospital* or "Inpatient setting*" or "Critical care" or "Emergency care" or "Intensive Care").ab. | 785655 |
| 10 | 8 or 9 | 811397 |
| 11 | 3 and 6 and 7 and 10 | 248 |

**Embase <1974 to 2024 May 14>**

| **Search** | **Query** | **Records retrieved** |
| --- | --- | --- |
| 1 | exp nurse manager/ or exp nurse administrator/ | 16127 |
| 2 | ("Nurs* manager*" or "Nurs* administrator*" or "Nurs* leader*" or "Charge Nurse*" or "Ward Manager*" or "Unit Manager*" or "Nurs* supervisor*").ti. or ("Nurs* manager*" or "Nurs* administrator*" or "Nurs* leader*" or "Charge Nurse*" or "Ward Manager*" or "Unit Manager*" or "Nurs* supervisor*").ab. | 15165 |
| 3 | 1 or 2 | 25789 |
| 4 | *evidence based practice/ or exp evidence based nursing/ | 17699 |
| 5 | ("Evidence-based pract*" or "Evidence-Informed pract*" or "Evidence-based healthcare" or "Evidence-based Nursing" or EBP or EBN or "Evidence implementation" or "research utili$ation").ti. or ("Evidence-based pract*" or "Evidence-Informed pract*" or "Evidence-based healthcare" or "Evidence-based Nursing" or EBP or EBN or "Evidence implementation" or "research utili$ation").ab. | 31573 |
| 6 | 4 or 5 | 45002 |
| 7 | (Factor* or Influence* or Facilitat* or Determinant* or Barrier* or Enabler* or Knowledge or Skill* or Attitude* or Perception* or Belief* or "Learning need*" or Practice or Support* or Promote* or Competenc*).ti. or (Factor* or Influence* or Facilitat* or Determinant* or Barrier* or Enabler* or Knowledge or Skill* or Attitude* or Perception* or Belief* or "Learning need*" or Practice or Support* or Promote* or Competenc*).ab. | 12822241 |
| 8 | hospitals/ or exp Acute services/ or exp Acute care/ | 334620 |
| 9 | ("Acute care" or Hospital* or "Inpatient setting*" or "Critical care" or "Emergency care" or "Intensive Care").ti. or ("Acute care" or Hospital* or "Inpatient setting*" or "Critical care" or "Emergency care" or "Intensive Care").ab. | 2872919 |
| 10 | 8 or 9 | 2977185 |
| 11 | 3 and 6 and 7 and 10 | 280 |

**Cochrane Register: Date Run-18/04/2024 08:13:10**

| **Search** | **Query** | **Records retrieved** |
| --- | --- | --- |
| #1 | MeSH descriptor: [Nurse Administrators] explode all trees | 41 |
| #2 | ((Nurs* NEXT manager*) OR (Nurs* NEXT administrator*) OR (Nurs* NEXT leader*) OR (Charge NEXT Nurse*) OR (Ward NEXT Manager*) OR (Unit NEXT Manager*) OR (Nurs* NEXT supervisor*)):ti,ab,kw (Word variations have been searched) | 412 |
| #3 | #1 or #2 | 412 |
| #4 | MeSH descriptor: [Evidence-Based Nursing] explode all trees | 91 |
| #5 | ((Evidence-based NEXT pract*) OR (Evidence-Informed NEXT pract*) OR (Evidence-based NEXT healthcare) OR (Evidence-based NEXT Nursing) OR "EBP" OR "EBN" OR (Evidence NEXT implementation) OR (research NEXT utili?ation)):ti,ab,kw (Word variations have been searched) | 4591 |
| #6 | #4 or #5 | 4591 |
| #7 | MeSH descriptor: [Acute Care Surgery] explode all trees | 38 |
| #8 | MeSH descriptor: [Critical Care] explode all trees | 3085 |
| #9 | MeSH descriptor: [Child, Hospitalized] explode all trees | 196 |
| #10 | MeSH descriptor: [Adolescent, Hospitalized] explode all trees | 10 |
| #11 | ((Acute NEXT care) OR Hospital* OR (Inpatient NEXT setting*)):ti,ab,kw (Word variations have been searched) | 247342 |
| #12 | #7 OR #8 OR #9 OR #10 OR #11 | 249061 |
| #13 | #3 AND #6 AND #12 | 9 |

**Scopus**

( TITLE ( "nurse manag*" OR "nurs* leader*" OR "nursing management" OR "nursing administration" OR "nurs* administrator*" OR "ward manager*" OR "charge nurse" OR "Nurs* supervisor*" ) OR ABS ( "nurse manag*" OR "nurs* leader*" OR "nursing management" OR "nursing administration" OR "nurs* administrator*" OR "ward manager*" OR "charge nurse" OR "Nurs* supervisor*" ) AND TITLE ( "evidence based practice" OR "evidence-based practice" OR "evidence based nursing" OR "research utilization" OR "research implementation" ) OR ABS ( "evidence based practice" OR "evidence-based practice" OR "evidence based nursing" OR "research utilization" OR "research implementation" ) AND TITLE ( barrier* OR enabler* OR facilitator* OR challenge* OR hinder* OR obstruct* OR constrain* OR promote* OR boost* OR drive* OR aid OR assist OR support OR leader* OR champion* ) OR ABS ( barrier* OR enabler* OR facilitator* OR challenge* OR hinder* OR obstruct* OR constrain* OR promote* OR boost* OR drive* OR aid OR assist OR support OR leader* OR champion* ) AND TITLE ( hospital OR "acute care " OR "inpatient setting" OR "critical care" OR "emergency care" OR "intensive care" ) OR ABS ( hospital OR "acute care " OR "inpatient setting" OR "critical care" OR "emergency care" OR "intensive care" ) )

**Results: 193, Limited to English: 190 Date: 15/5/2024**
