# Supplementary material for: Understanding, Using, and Facilitating Evidence-Based Practice: A Scoping Review of Influencing Factors Among Nurse Managers in Acute Care
Source: J Nurs Manag. 2025 Jul 4;2025:2155376. doi: 10.1155/jonm/2155376 (PMC12253997; doi:10.1155/jonm/2155376)
Supplement: Supporting Information 2 — Appendix 2: Data extraction instrument.docx. [file 2155376.f2.docx]

### Appendix II: Data extraction instrument

| **Study ID**  **Author(s)**  **Year of publication**  **Country of study** | **Methodology /**  **Method** | **Participants** **Characteristics and sample size** | **Enablers** **to Understanding and Use of EBP** | **Barriers** **to Understanding and Use of EBP** | **Interventions and Strategies /**  **Recommendations** | **Strengths** | **Limitations** |
| --- | --- | --- | --- | --- | --- | --- | --- |
|  |  |  |  |  |  |  |  |
|  |  |  |  |  |  |  |  |
|  |  |  |  |  |  |  |  |
|  |  |  |  |  |  |  |  |
|  |  |  |  |  |  |  |  |
|  |  |  |  |  |  |  |  |
|  |  |  |  |  |  |  |  |
