# Supplementary material for: Understanding, Using, and Facilitating Evidence-Based Practice: A Scoping Review of Influencing Factors Among Nurse Managers in Acute Care
Source: J Nurs Manag. 2025 Jul 4;2025:2155376. doi: 10.1155/jonm/2155376 (PMC12253997; doi:10.1155/jonm/2155376)
Supplement: Supporting Information 3 — Appendix 3: Extracted data for the study.docx. [file 2155376.f3.docx]

| **Study ID**  **Author(s)**  **Year of publication**  **Country of study** | **Methodology /**  **Method** | **Participants** **Characteristics and sample size** | **Enablers** **to Understanding and Use of EBP** | **Barriers** **to Understanding and Use of EBP** | **Interventions and Strategies /Recommendations** | **Strengths** | **Limitations** |
| --- | --- | --- | --- | --- | --- | --- | --- |
| (Gallagher-Ford et al., 2023)  United States | Cross-sectional design using survey methodology.  Survey conducted via Qualtrics, anonymous responses implied consent., recruited via email, AMSN and MSNCB websites, and social media.  Descriptive statistics used for data analysis.  EBP attributes measured using valid and reliable instruments developed by Melnyk et al.​ | The participants were members of the Academy of Medical-Surgical Nurses (AMSN) or certified medical-surgical RNs.  Total sample size was 1709 nurses, with 47% (n=798) being clinical staff nurses, 28% (n=482) midlevel leaders, 11% (n=185) nurse educators, and 3% (n=46) senior leaders.  Surveys sent to approximately 13,000 nurses with a response rate of 13%​. | Significant positive effects on EBP implementation included high EBP competencies, high EBP beliefs, senior leaders, nurses with an MSN or higher, and those in organizations with EBP recognition.  “High implementation was predicted by a combination of high EBP beliefs scores and high EBP competency scores.”  Highest implementation rate included “High EBP beliefs and high EBP competencies, and working in a PRISM unit” | Barriers to EBP implementation included lack of prioritization, insufficient resources, lack of understanding, lack of confidence, and limited time.  Lack of time for EBP was the most identified barrier across all role groups.  Lack of understanding of EBP and lack of confidence in implementing EBP.  Contextual factors affecting EBP implementation include organizational culture, leadership support, and insufficient resources.  “Low EBP implementation was predicted by having a low EBP competency score regardless of EBP belief score” | N/A  Recommendation:  Leaders should prioritize and protect time for evidence-based practice (EBP) across the organization, using appropriate language to reflect its value. Senior leaders must build EBP infrastructures, including incorporating it into career development and performance appraisals, and identifying EBP champions. Mid-level and senior nursing leadership should develop a culture of EBP, build EBP competency, and remove barriers to EBP implementation. Nurses require dedicated, paid time to build EBP knowledge and skills, as well as access to resources and support for EBP certification. Senior leaders without advanced education should consider pursuing it to improve EBP competencies and leadership. | The strengths of the study included valid and reliable instruments, a large sample size allowing for comparisons between role groups and education levels. | Low response rates, potential selection bias, focus on medical-surgical nurse leaders, potential self-selection bias, limited generalizability, reliance on self-report measures, and lack of objective measurements. |
| (Wilkinson et al., 2011)  Scotland, UK | A qualitative case study approach using interviews, documentary analysis, and observational data. | The study included Nurse Managers (NMs), Nurse Directors, Practice Development Nurses, Charge Nurses, Clinical Educators, and Clinical Nurse Specialists across four Scottish Health Boards.  The total sample size was 51 participants. | Presence of Nurse Directors and NMs championing EBP. ​  NMs empowering and facilitating nurses to engage with EBPI, linking appropriate people to EBPI posts)  Stable nursing workforce.  Well-connected Practice Development Units (PDUs), clear lines of communication and integration of EBP initiatives. | Overloaded roles and competing demands on nurses' time.  Lack of clarity about roles and responsibilities for evidence-based practice implementation (EBPI).  Limited/Passive NM involvement in EBPI.  Limited support and autonomy for charge nurses to make changes to practice. ​  Insufficient knowledge and skills among NMs to support EBPI. ​  Poorly connected Practice Development Units (PUDs) and organizational restructuring challenges.  Perceived management-practice gap, where management was seen as increasingly generalized and remote from practice. | Suggestions for improvement:  Nurse Directors and NMs need to actively champion and support EBP. ​  NMs should play a linking role in integrating EBP initiatives and linking staff to EBPI posts. ​  NMs should provide support, empowerment, and autonomy for nurses to engage with EBPI. ​  NMs should have a more visible presence in clinical areas to demonstrate interest in practice. | Detailed exploration of the roles of NMs in EBPI. ​  Identification of contextual factors that explain the limited engagement of NMs in EBPI. | Case study research provides contextualized findings but cannot be generalized. |
| (Camargo et al., 2018)  Brazil | Qualitative research guided by the Theory of the Diffusion of Innovations  Five focus group workshops were conducted, and textual records resulting from each workshop were analysed by semantic categories. | Nurse managers from a Teaching Hospital in Triângulo Mineiro, Brazil. ​  The sample size was 18 participants. | Timely access to the results of research conducted at the teaching hospital was mentioned as crucial to guide better practices. ​  Engagement of Nursing coordination and leadership was identified as a crucial strategy to make the hospital context favourable to EBP. ​  The engagement of opinion leaders who encourage others to adopt innovation was seen as important in the diffusion of EBP.  Access to resources like computers with internet and safeguarded study time were identified as enablers​  Interest in "questioning and opening to the new" was identified as essential for implementing EBP. | Limited financial resources.  Hierarchical structures unclear nurse manager role, and fragmented work practices hindered team integration and motivation for EBP.  The improvised act of the nurse, although sometimes leading to the discovery of new techniques, was seen as a barrier to the systematic use of evidence. ​  Lack of knowledge and ability to analyse research, and limited knowledge sharing by researchers leading to a sense of alienation and lack of engagement with research were identified as barriers to the development and utilization of research. | Workshops using a hermeneutic-dialectic approach.  Recommendation:  Further research is needed to develop strategies to address the identified barriers and enhance the enablers for successful EBP implementation. | The hermeneutic-dialectical approach provided deep qualitative insights into the experiences of nurse managers. | The study was context-bound to the teaching hospital limiting generalization to other healthcare settings. |
| (Camargo et al., 2016)  Brazil | Descriptive-quantitative study evaluating workshops conducted with nurse leaders aimed at promoting EBP.  The intervention and its evaluation were guided by the conceptual models of Rogers for Diffusion of Innovations and Melnyk, Gallagher-Ford & Fineout-Overholt for Competencies for Evidence-Based Nursing Practice in Care Settings. | Nurse managers from a Teaching Hospital in Triângulo Mineiro, Brazil.  The sample size was 18 participants. | Valuing opportunities to discuss real-world challenges and interact with their peers during workshops.  Positive attitudes toward incorporating evidence into practice.  Being open to questioning about their practice.  Ability to formulate a question  Ability to review their own practice. | Difficulty in understanding research methods and critical analysis of scientific articles.  Lack of knowledge on how to search for evidence  Lack of time dedicated to seeking new evidence in their work agenda. | Conducted workshops focused on EBP addressing practical aspects of incorporating research findings:  Ensured access to scientific databases and journals, engaged hospital leadership in promoting and supporting EBP and provided nurses with designated time for engaging with and applying EBP, and encouraged a cultural shift within healthcare settings to value and prioritize EBP.  **Recommendation:**  Invest and equip NMs with leadership skills beyond competence to foster evidence-based practice, such as transformational leadership.  Future research should examine group behaviour, competency acquisition, EBP barriers and NMs role in EBP. | Innovative approach targeting nurse managers  Positive impact on motivation and understanding of EBP  The qualitative approach allowed for a detailed understanding of the nurse managers' perspectives. | The small sample size and the need for further research to assess long-term impact were limitations. |
| (Shuman et al., 2018)  USA | A multi-site, multi-unit cross-sectional design was used.  Surveys were administered electronically via Qualtrics to nurse managers and staff nurses. | The study included nurse managers and staff nurses from different-sized hospitals representing different regions in the USA.  The sample size was 24 nurse managers and 264 randomly selected staff nurses from 24 adult medical-surgical units from 7 acute care hospitals in the Northeast and Midwestern USA. | Positive EBP leadership behaviours (Proactive, knowledgeable, supportive, and perseverant) of nurse managers significantly contributed to a supportive unit climate for EBP implementation.  Higher levels of EBP competency among nurse managers significantly contributed to a positive climate for EBP implementation | Significant variability in unit climates for EBP implementation existed across different hospitals and units, indicating inconsistent support, recognition, and resources for EBP.  More years of experience in the current unit showed a significant but potentially limiting factor on the positive climate for EBP, suggesting that longer tenure might relate to resistance to change or adoption of new practices. | **Recommendation:**  Future studies should use experimental designs to test interventions that target nurse manager leadership behaviours and competencies to improve unit climates and EBP implementation.  More research is needed to further explicate the relationships among nurse manager leadership, unit climate, and other implementation outcomes in various care settings. | Robust data collected from multiple sites and a large, diverse sample.  Nurse managers, often overlooked, were targeted to understand their role in EBP.  Diverse sample enhances generalizability of findings.  Reliable scales measured leadership and climate.  Regression analysis examined relationships between manager behaviours, climate, and EBP implementation. | The study’s cross-sectional nature limited the ability to infer causality between nurse manager behaviours, competencies, and EBP implementation climate.  Reliance on self-reported measures from participants may introduce bias or inaccuracies in the data.  Limited generalizability as hospitals were conveniently selected |
| (Lai et al., 2022)  China | Descriptive study employing a regional cross-sectional survey.  Online questionnaire survey conducted via email.  Hematic analysis was conducted to analyse the responses to the open-ended question | Nursing directors or coordinators from 79 tertiary hospitals and 10 secondary hospitals in Guangdong Province, China.  The respondents included 73 nursing directors and 16 nominated nurses responsible for conducting EBNP in their respective hospitals. | N/A  However, it suggests that hospital nursing managers play a pivotal role in promoting evidence-based nursing practice, especially in the Chinese context. | Lack of knowledge about the steps of evidence-based nursing practice, literature search, critical appraisal of evidence, and data analysis.  Lack of history of innovation and change, lack of EBNP expert and team support, inadequate human resources, lack of EBNP training opportunities, and insufficient time and heavy workload.  Resistance to adopting EBNP due to adherence to traditional practices and Lack of collaboration between departments and health workers.  Low prioritization of EBNP in the hospital's strategic agenda and lack of intention to implement EBNP among nurses.  Lack of confidence among nursing staff in their ability to perform EBNP effectively. | Recommendation:  Hospitals should provide systematic EBNP education and training for both clinical nurses and nursing managers to improve their EBNP knowledge, beliefs, and skills.  Hospitals should strengthen the EBNP culture and organizational support, such as identifying incentives, funding, and establishing EBNP education courses.  Universities should incorporate EBNP as a compulsory course in undergraduate nursing education.  Introducing clinical librarians into hospital teams could help nurses adopt EBNP. | High response rate of 100% for the questionnaires sent out and a large sample size of 89 hospitals across Guangdong Province, providing a broad perspective on the barriers to EBNP.  Thematic analysis of responses to the open-ended question provides detailed insights into the barriers to EBNP. | The study focused primarily on identifying barriers to EBNP rather than exploring enablers or effective strategies in detail.  The study was limited to hospitals in Guangdong Province, which may not be representative of other regions in China or globally. |
| (Kitson et al., 2011)  Australia | Focused ethnographic approach.  Data were collected through questionnaires, interviews, focus groups, and observations of clinical nursing leaders, team members, and service managers over a 12-month intervention period​ to understand the implementation of evidence-based practice at a local level​. | 14 volunteer clinical nursing leaders ​  28 team members from the seven KT improvement teams  11 service managers (7 nursing managers and 4 medical managers) | Managers recognized the importance of local level innovation and integrating local priorities with broader system-wide agendas.  Clinical nursing leaders were motivated by the opportunity to network, be involved in something innovative, and improve care for older people  Clinical nursing leaders could choose projects that aligned with their strengths, fostering commitment.  Psychological safety and ongoing support were critical for enabling local leaders.  Acknowledgment of the power and authority of positional leadership roles facilitated clinical improvements.  Working with allied health professionals and networking across the organization were seen as positive  Selecting focused areas for implementation and improvement was crucial​.  Observation of increased confidence, teamwork skills, and "can-do" attitudes in clinical nursing leaders | Clinical nursing leaders expressed anxieties and risks associated with taking on a leadership role in implementing change and unpreparedness for leading interdisciplinary, cross-functional teams.  Maintaining momentum and involvement was challenging due to time pressures (length of stay, multiple comorbidities, siloed working.)  Resistance to change to traditional, siloed work practices and the inability to see the bigger strategic picture.  Lack of cohesion and integration between professional groups and the complexity of group dynamics led to conflicts and personalization of problems.  Cynicism about organizational commitment.  Many clinical nursing leaders lacked experience in formal audits and evidence implementation mechanisms.  Barriers such as poorly designed processes, resource constraints, and non-professional duties impacted care delivery and innovation to the desired standards of care​.  Lack of time | Structured implementation program with a KT Toolkit to support clinical nursing leaders to improve care for older people across seven clinical areas.  Provision of support and "psychological safety" for clinical nursing leaders to experiment and innovate:  -evidence implementation and dissemination.  -Encouraged leader volunteerism to increase commitment.  -Provided ongoing support and a safe environment for innovation.  -Fostered interdisciplinary collaboration.  -Equipped leaders with skills and knowledge through structured training.  -Prioritized holistic, patient-centred care.  **Recommendation:**  Further studies to understand the role of managers in facilitating knowledge translation initiatives and creating a supportive environment for local leaders to experiment and innovate.​ | Ethnographic approach allowed for in-depth understanding of the cultural and contextual factors as well as exploration of the experiences of the key stakeholders (clinical nursing leaders, team members, and service managers)  Semi-structured interviews and questionnaires, providing rich insights into the participants' experiences.  The study highlighted real-world challenges and successes in implementing evidence-based practice within a clinical setting​ | The sample size of the clinical nursing leaders was relatively small (14 participants), which may limit the generalizability of the findings.  The study was conducted in a single hospital, which may limit the generalizability of the findings to other healthcare settings.  The use of manual notetaking during interviews, rather than audio-recording, may have resulted in less detailed transcripts. |
| (Renolen et al., 2020)  Norway | A classic grounded theory methodology to generate a substantive theory about patterns of leader behaviour that leaders are engaged in when attempting to integrate evidence-based practice (EBP) in a clinical setting.  Data collection was via observations, individual interviews, and focus groups these were collected over a period of four years, from March 2014 to January 2018 to develop a theoretical framework based on the data collected. | The participants included 15 specialist nurses, 39 registered nurses, and 9 assistant nurses.  All participants were observed, 5 ward leaders (2 head nurses, 1 assistant head nurse, and 2 teaching nurses) participated in individual interviews, and 18 clinical nurses participated in 4 focus groups.  The specialist nurses had education beyond basic nursing education equivalent to 60 ECTS credits, with two having 120 ECTS credits.  All leader participants had completed EBP seminars years before the study but could not recall specific content. | Leaders leveraged their experience and prior EBP training to drive implementation.  Nurse managers shared resources, knowledge, and engage in regular team meetings to foster EBP by enhancing structure, strategically dividing tasks, and following up on clinical issues.  Managers provided practical assistance to nurses to overcome challenges, such as adjusting workloads, modifying routines, offering dedicated EBP time, and being readily available.  Nurse managers inspired and motivated nurses to use EBP and national guidelines in their practice and creating a space for them to ask questions and encourage EBP discussions.  Nurse managers assessed nurses' EBP interest and performance, gathered feedback, and analysed the consequences of EBP integration and provided feedback. | Lack of time, capacity, staff, and proper infrastructure (e.g. access to computers and optimal working spaces) to effectively implement EBP.  Organizational Culture focused on standardized routines and task completion leaving little room for EBP activities.  Pressure to complete standardized routine procedures over addressing individual patient needs  Nurses already having a heavy workload with too many check offs and reports with limited time for professional teaching activities or seminars and to integrate EBP activities.  Nursing leaders having limited opportunities to observe nurses' daily work, making it difficult to identify and address challenges related to EBP integration.  Focus on allocating tasks to nurses rather than facilitating teamwork and learning.  Managing interdisciplinary teams and group dynamics presented challenges. | Recommendation: Develop and implement training programs focused on leadership skills that support EBP.  Increase Organizational Support by ensuring adequate resources and support systems are in place to facilitate EBP activities.  Foster a culture of collaboration and teamwork to enhance the implementation of EBP.  Provide protected time for nurses to engage in EBP activities without compromising their clinical duties​.  Future research should explore the interactions between leaders and nurses in the EBP integration process to further enhance the leaders' knowledge and ability to adjust EBP integration to clinical practice.  Engagement and interactions within a leader team seem to have enabled the EBP integration process, and this area warrants further investigation. | Use of multiple data collection methods (observations, interviews, focus groups) provided rich, detailed information.  The application of grounded theory allowed for the development of a robust theoretical framework based on empirical data.  Extended data collection period (four years) enabled a thorough understanding of the implementation process. | Limited sample size may affect the generalizability of the findings.  Lack of detailed information about the content of EBP seminars attended by leaders |
| (Weng et al., 2016)  Taiwan | Cross-sectional and longitudinal surveys were conducted nationwide among Directors of Nursing (DONs) in regional hospitals in 2007, 2009, and 2011 to examine their views related to Evidence-Based Practice (EBP). The survey measured changes in beliefs, attitudes, knowledge, skills, behaviours, and barriers. | The study enrolled 267 DONs in 2007, 257 in 2009, and 287 in 2011. All participants were female, with an average age of 37.9, 38.8, and 38.8 years and an average working period of 14.8, 15.5, and 15.5 years, respectively. | Beliefs and attitudes towards EBP were positive with nurse leaders viewing EBP as important and willing to support implementation throughout the years.  Increased knowledge and skills in EBP over time.  Increased use of Internet-based resources such as web portals, electronic textbooks, electronic journals, and evidence-based online databases. | Most barriers to EBP implementation, such as lack of capable personnel, convenient application kits, time for EBP, basic knowledge of EBP and skills in critical appraisal, support from superiors, and literature searching, and library resources, especially having literature sources in the Chinese language, significantly declined after the intervention. However, the lack of clinical incorporation became more prominent as a barrier.  Their attitudes to EBP did not change over time despite their knowledge and skills increasing | Implementation of multifaceted interventions, including workshops, conferences, and the use of a professional organization (TEBMA) for mass media campaigns to disseminate EBP​:  Workshops included training in research methods, literature searching, critical appraisal, and clinical decision-making.  Provision of information support and access to Internet-based resources to help use evidence​:  The program provided EBP-related information resources and promotional activities.  Access to a Chinese-language website for EBP information and free usage of the Cochrane Library.  Translation of Cochrane Database of Systematic Reviews abstracts into Traditional Mandarin Chinese.  Hosting annual conferences, regional hospital workshops, and EBP contests to enhance engagement and knowledge  **Recommendation:**  Spreading EBP through a mass media campaign may serve as a key element to accelerate its implementation. In addition, continued emphasis on educational interventions for DONs to enhance their knowledge and skills in EBP is needed.  Organizational support and provision of resources to overcome barriers such as time constraints and lack of personnel.  Further research to explore additional strategies to foster support for EBP among DONs to overcome the challenge of incorporating EBP into clinical practice and evaluate long-term impacts on patient care quality. | Longitudinal data collection over five years, allowing for the observation of trends and changes in EBP implementation  Nationwide representative sample providing comprehensive insights into the dissemination of EBP among DONs in regional hospitals.  Use of validated survey instruments with high content validity and reliability. | Self-reported data which may introduce response bias.  The study focused exclusively on female DONs, limiting the generalizability to male DONs (I suspect there is none or very limited in Taiwan)  The questionnaires were conducted at three points in time, which may not fully capture the ongoing changes in EBP implementation  Respondents did not overlap completely across the three questionnaire surveys, potentially affecting consistency​. |
| (Royle et al., 1997)  Canada | Cross-sectional survey design utilizing a mailed questionnaire to gather data. The questionnaire aimed to assess existing information resources, the information management skills of nurses, and the additional resources and training required. | The sample consisted of 67 out of 71 (94%) nursing administrators from all hospitals in Central West (52) and Northwest Ontario (19) completed the questionnaire.  But 65 questionnaires were usable for analysis. The hospitals varied in size, with annual patient admissions ranging from 57 to 550,000, and the number of beds ranging from fewer than 25 to over 500. | Accessibility of libraries and electronic bibliographic databases, availability of interlibrary loans, and connections to health sciences library networks in larger hospitals​  Orientation and educational courses in information management skills provided in some hospitals.  Existing research/ Nursing Quality Assurance/Improvement committees with nursing representation, nursing research directors, and clinical nurse specialist particularly in larger hospitals. | Smaller hospitals often lacked librarians, library employees, and electronic resources.  Limited access to nursing journals in smaller hospitals.  Inconsistent provision of training and orientation related to research utilization and information management skills.  Time constraints and lack of access to research literature.  Variability in organizational environments and their conduciveness to accessing and retrieving research information.  Financial constraints prohibiting the establishment of courses  A perceived lack of appreciation among staff nurses for literature search and retrieval skills. | **Recommendations:**  There is a need for better information resources and skills to access and evaluate professional literature among nurses.  Nursing administrators should work to minimize barriers and help staff nurses recognize the importance of information management skills for professional development and patient care.  Development of distance education programs to address training gaps  Use of technology to make services and resources accessible to nurses in smaller hospitals.  Establishment of partnerships and cooperative ventures to share resources and expertise across hospitals | High response rate (94%) from hospitals, providing a comprehensive overview of the information resources and needs in the regions studied.  Detailed analysis of the variations in resources and training opportunities across different sizes of hospitals. | The study was limited to two regions in Ontario, which may not be representative of other regions or countries.  The data relied on self-reported questionnaires, which could be subject to response bias. |
| (Johansson et al., 2010)  Sweden | Quantitative survey using a web-based questionnaire.  Data were collected using a web-based questionnaire developed specifically for the study, inspired by the Research Utilization Questionnaire (RUQ). | All 168 head nurses at a university hospital and a county hospital in Sweden were invited to participate.  Sample size: 99 head nurses (59%) completed the survey. | Positive attitude towards EBP.  More years as a head nurse correlated with greater research utilization.  Education in research methods significantly associated with increased EBP activities.  Perceived support from immediate superiors emphasizing the importance of EBP significantly associated with increased EBP activities. | Lack of time for EBP activities, such as reading research reports and conducting research during work hours.  Head nurses seldom discuss research findings with staff members.  Insufficient time for staff to search for and read research findings. | An EBP course was conducted at Uppsala University Hospital.  The course consisted of five full educational days over 15 weeks.  Included topics: EBP concepts, literature search, reading scientific papers, data collection methods, basic data management, and presentation of development projects.  Supported by hospital research and development leaders during the course.  Encouragement and support from immediate superiors to attend the EBP course and complete development projects during work hours.  Recommendations:  Education in EBP and research methods should be prioritized for head nurses.  Supportive leadership is crucial for the successful implementation of EBP.  Interventions to provide more time for EBP activities by head nurses are needed.  Management should create strategies to enhance support for EBP, aiming to deliver high-quality care and increase patient satisfaction.  Conduct further studies to explore the long-term effects of educational interventions on EBP adoption and sustainability. | High response rate (59%) from head nurses.  Comprehensive survey covering various aspects of EBP.  Clear positive correlation between education, support from superiors, and EBP activities.  Highlighted the pivotal role of head nurses in the development of EBP. | Limited to two hospitals, which may affect generalizability.  Self-reported data may be subject to bias.  Cross-sectional design limits causal inferences.  Potential influence of unmeasured confounding factors. |
| (Kueny et al., 2015)  USA | Qualitative descriptive design using interviews | Nine nurse managers (NMs) from high- and low-performing units, all female, with an average of 6.5 years of experience. | A culture that values and encourages the use of evidence-based practice (EBP) (e.g. setting expectations and evaluations for NM and staff focused on EBP, institutional appreciation for involving staff in decisions, and collaboration or teamwork among the managers of different units).  Transparent and consistent communication about the importance and goals of EBP.  Regular interactions with top management to align EBP initiatives with organizational goals.  Dedicated committees focused on nursing practices and EBP.  Availability of resources such as quality-monitoring departments to support EBP initiatives.  The process of obtaining Magnet status | Insufficient backing from higher management for EBP  Unclear or ineffective methods to encourage nurses to adopt EBP.  Limited access to necessary resources and tools to implement EBP effectively. | N/A  **Recommendations:**  Strengthen the support from higher management to foster a culture that prioritizes EBP.  Create and implement clear, effective strategies to motivate nurses to engage in EBP.  Ensure that nurse managers have adequate access to the necessary resources and tools to support EBP initiatives.  Continued research to refine the measurement of institutional context and its impact on EBP implementation. | Provides in-depth understanding of institutional contextual factors affecting NMs’ ability to implement EBP  Compares perspectives of nurse managers from high-performing and low-performing units. | Small sample size (nine NMs), limiting generalizability. |
| (Barako et al., 2012)  Kenya | Cross-sectional descriptive study: Utilized questionnaires and interview guides to collect quantitative and qualitative data respectively. | Of the 156 nurses recruited, 144 (92.3%) returned surveys. This included 14 nurse managers who were also interviewed. Nurses were from medical, surgical, SOPC, and MOPC wards. | Supervision of care  Providing updates to staff  Promoting use of guidelines | Lack of supplies, equipment and EB guidelines.  Staff shortages  Time constraints  Inadequate understanding of EBP | Recommendations: Develop more nurse graduates, expose nurses to research courses, and improve resource availability.  Management embracing the importance of EBP and providing the necessary support in terms of resources and other infrastructures. | Included both quantitative and qualitative methods with a high response rate of 92.3%. | Unclear findings specific to nurse managers from quantitative data |
| (Farokhzadian et al., 2015)  Iran | A cross-sectional study conducted using a modified version of the "perceptions of nurses of evidence-based practice questionnaire". | Study included 70 nurse leaders from four teaching hospitals in Kerman. Majority were female (81.4%), aged 35-45 years, with over 10 years of nursing experience | Positive attitudes and higher self-efficacy were moderately correlated, indicating that those with positive attitudes felt more confident in EBP activities. | Unfavourable attitudes towards EBP, low self-efficacy in EBP skills, high workload, lack of specific training, and minimal participation in research activities. | **Recommendation:**  Recommend enhancing training programs and support for nurse leaders to improve EBP implementation, revising curricula, and developing strategies to promote EBP in healthcare systems.  Nurse leaders need to equip themselves with the necessary attitudes and skills to promote evidence-based practice. | High response rate (74%), comprehensive assessment of attitudes, self-efficacy, and training needs. | Limited to four hospitals in one region which may not be generalizable. |
| (Patton et al., 2024)  USA | Quantitative, pre-test-post-test comparative design | Nursing Leaders: 34 participants, predominantly from critical care units, with over 11 years of nursing experience and less than three years of nursing leadership experience.  Clinical Nurses: 51 reporting to the participating nursing leaders. | Participation in an Evidence-Based Practice (EBP) Leadership Behaviour Program improved EBP competencies and leadership behaviours:  Improvement in EBP competencies and behaviours  Supportive leadership behaviours (e.g., recognizing and supporting EBP efforts) | Nursing leaders exhibited low EBP competencies and low EBP leadership behaviours, particularly in the proactive leadership domain, prior to the intervention.  Clinical nurses perceived their nursing leaders as lacking in proactive EBP leadership behaviours and this did not improve post-intervention | A 12-hour EBP Leadership Behaviour Program with workshops and activities led by EBP experts  EBP Leadership Behaviour Program (LBP) consisting of workshops, synchronous/asynchronous activities, and guided EBP process  Emphasis on developing leadership behaviours (proactive, supportive, knowledgeable, perseverant) to facilitate EBP implementation  Recommendation:  Advocacy for leadership development programs tailored to EBP competencies  Emphasis on creating organizational climates supportive of EBP through proactive leadership and resource allocation. | Use of established frameworks (Ottawa Model of Implementation Leadership, Implementation Science)  Rigorous quantitative design with pretest-post-test measurements  Large effect sizes observed in improvements among nursing leaders | Limited generalizability beyond the studied healthcare system and specific types of nursing units.  Reliance on self-reported measures for nursing leaders and clinical nurses' perceptions  Lack of significant improvement in clinical nurses' perceptions of leadership behaviours post-intervention (Timing of data collection and intervention delivery may have influenced results.) |
| (Hasanpoor et al., 2019)  Iran | This study used a cross-sectional descriptive design and collected data through a self-administered questionnaire. The questionnaire was developed based on a review and Delphi studies, identifying facilitators and barriers to Evidence-Based Management (EBMgt). | A total of 276 nursing managers from 20 hospitals were invited to participate in the study, and 212 nursing managers responded, reflecting a response rate of 76.81%.  Most participants were head nurses and supervisors, and 63.2% had a bachelor's degree. ​ The mean age of participants was 41.34 years, and the average work experience was 17.17 years. | Enablers included organizational support, manager characteristics and individual factors, research production factors, external or environmental factors, and social or interpersonal factors. The highest scores were for "interest and willingness to scientific management principles"​  study indicated that organizational support, existence of adequate resources, and use of continuing education programs facilitated the implementation of EBMgt for nursing managers. | The barriers to EBMgt were categorized into five main domains: decision-maker characteristics, decision-making environment, training and research systems, organizational barriers, and team barriers. The highest mean scores for barriers were found in the domain of "training and research systems."  The highest scores across the domains were for "lack of communication between knowledge producers and hospital decision-makers," "limits of innovation and new ideas in hospital administration," "organizational hierarchy and high bureaucracy," and "insufficient workforce" respectively. | Recommendation included continuing education for nursing managers, creating a favourable environment, providing resources and time for EBMgt, and fostering interaction between knowledge producers and nursing managers to ensure relevant and evidence-informed practice​ | The study's strengths include a high response rate and the use of a comprehensive questionnaire that covers a wide range of barriers and facilitators to EBMgt. It also provides practical recommendations for improving the implementation of EBMgt in healthcare settings.  **The study provided insights into the perspectives of nursing managers on the facilitators and barriers to EBMgt. ​** | The study's limitations include its cross-sectional design, which does not allow for causal inferences. Additionally, the study is limited to nursing managers in Iran, which may limit the generalizability of the findings to other contexts and countries​ |
| (Almaskari, 2017)  Sultanate of Oman | The study employed a descriptive, cross-sectional design using questionnaires to gather data on the attitudes of Omani staff nurses and nurse leaders towards evidence-based practice  The study used two valid and reliable instruments - the Evidence-Based Nursing Attitude Questionnaire (EBNAQ) and the Developing Evidence-Based Practice (DEBP) questionnaire. | The study involved 260 Omani nurse subjects, consisting of 162 staff nurses and 98 nurse leaders, all working full-time in different units of three government hospitals in Northern Oman (Nizwa, Ibra, and Sur hospitals). The sample comprised 82.7% females (n=215) and 17.3% males (n=45) with a mean age of 32 years. | Commitment to advancing the nursing profession.  Support and encouragement from nursing administrators.  Positive attitudes towards EBP among participants.  There were no significant differences in the attitudes towards EBP between the two groups. | Workload and time constraints.  Lack of doctoral programs in nursing in Oman.  The need for more resources and training in EBP.  The study found that nurse leaders perceived more barriers to finding and reviewing evidence compared to staff nurses. However, there were no significant differences between the two groups in the barriers to changing practice and facilitators to changing practice. | No intervention  **Recommendation:**  Enhance EBP training and resources for nurses in Oman.  Establish doctoral programs in nursing to further advance the profession.  They suggest that continuous professional development programs should include components that promote EBP.  Nursing administrators should provide more support to reduce the workload and facilitate the implementation of EBP among nurses.  Conduct further research to explore other factors influencing EBP implementation and address the identified barriers. | High response rate (92.2%) from participants, which enhances the reliability of the findings.  Use of validated instruments like the Evidence-Based Nursing Attitude Questionnaire (EBNAQ) and Developing Evidence-Based Practice (DEBP) questionnaire.  Strong commitment from participants and support from hospital administrators. | Use of convenience sampling, which may limit the generalizability of the findings.  The modification of one item in the EBNAQ could have impacted its validity. |
| (Lynn & Moore, 1997)  USA | A Cross-sectional survey methodology was used. Nurse managers were asked to complete a slightly modified version of the BARRIERS tool, a 29-item instrument designed to measure perceived barriers to research utilization. | Sample Size: 40 nurse managers from seven hospitals  Demographics: Predominantly female (95%), 15% were enrolled in master's degree programs, and 34% held master's degrees in nursing or other fields. The median age was 42 years. 60% of participants had assisted with a research project, 50% had conducted research, and 36% were currently involved in some sort of research effort.  Sample Size: 40 nurse managers from seven hospitals (three urban, two rural, one academic medical centre, one federal hospital). | The study did not explicitly identify enablers, but it found that nurse managers who were currently involved in research projects perceived fewer barriers related to research values, skills, and awareness. | Difficulty in acquiring and understanding research  Limited time to read and implement research within job demands  Lack of support from nursing administration for research involvement  Barriers related to educational background, with diploma and associate degree-prepared managers perceiving more barriers  Rural hospital managers perceiving more barriers in research skills and awareness compared to those in urban settings  Presentation and accessibility of research findings  The highest rated barriers were related to the acquisition and understanding of research, such as the research literature not being readily available or understandable, and a lack of time to read and implement research. Barriers related to the perceived value and benefits of research for practice were rated as the lowest. | Recommendations:  Researchers should improve communication of research findings and presenting findings clearly and concisely.  Administrators need to support nurse managers by providing time and resources for research activities.  Educational programs should target nurse managers, particularly those with less formal education and those in rural settings, to enhance their research skills.  Nursing curricula should integrate research education across all courses, not just in designated research methods classes.  Creation of a supportive research environment in clinical settings is crucial for promoting research utilization. | Identification of specific barriers to research utilization among nurse managers  Comparison with previous studies to highlight changes over time  Use of a validated tool (BARRIERS tool) for measuring perceived barriers | Small sample size (40 nurse managers) with a limited geographic scope (seven hospitals)  Potential bias due to self-reported data  Lack of diversity in the sample, predominantly female participants |
| (Mathew et al., 2024)  United States of America | A mixed-method study with a sequential explanatory design.  It involved a national correlational survey using the Implementation Leadership Scale (ILS) and descriptive statistics for quantitative data collection and analysis, followed by qualitative data collection through semi-structured interviews. Gathered data was integrated to interpret the quantitative and qualitative data. | The study targeted Magnet-recognized hospital nurse managers (MRHNMs) with direct supervision of bedside nurses in critical and acute care units across 548 US Magnet-recognized hospitals.  The sample size for the quantitative phase was 153 MRHNM determined by a priori power analysis.  Twelve (12) MRHNMs were interviewed in the qualitative phase.  The participants were mostly females (83%) and Whites (56%), with an average age of 45 years.  The MRHNMs had a span of control of at least 40 direct reports and at least one year of employment in their current position. | MRHNMs viewed EBP as a healthcare priority and recognized the leadership of NMs as necessary to promote EBP.  Magnet Culture and Organizational Support with structured environment for EBP implementation and availability of resources such as research teams, EBP councils, and committees.  Doctoral nurses guide and support research and EBP, providing necessary knowledge and facilitating EBP projects.  Professional Development Opportunities including nurse residency programs with EBP projects. | A significant lack of knowledge about EBP among nurse managers and difficulties with the EBP process.  Time constraints and staffing issues were identified as barriers to implementing EBP among MRHNMs. | Recommendation:  There is a need for continuous education and training programs to improve nurse managers' knowledge and skills in EBP.  Strengthening organizational support and providing adequate resources are crucial for effective EBP implementation.  Promoting collaboration between nurse managers and doctoral-prepared nurses can facilitate EBP understanding and application. | The combination of quantitative and qualitative data provided a comprehensive understanding of the role of nurse managers in EBP.  National Scope: The study included multiple Magnet-recognized hospitals across the United States, enhancing the generalizability of the findings. | The reliance on self-reported data from surveys and interviews may introduce bias.  The study focused only on Magnet-recognized hospitals, which may limit the applicability of findings to non-Magnet hospitals. |
| (Gallagher-Ford, 2012)  USA | This research employed a descriptive correlational and comparative design. Data were collected using a demographic questionnaire and three validated instruments measuring EBP beliefs, perceptions of organizational readiness for EBP, and EBP implementation. | The sample consisted of 269 registered nurses, including 188 staff nurses, 32 nurse educators, and 49 nurse leaders from three hospitals in the United States. | Belief in the value and importance of EBP was a significant enabler for its implementation.  Positive perceptions of organizational readiness and support for EBP.  Support and influence from nurse leaders and educators played a crucial role in promoting EBP among staff nurses.  The study found that for both staff nurses and nurse educators, their EBP beliefs and perceptions of organizational readiness for EBP were significantly correlated to their implementation of EBP. | Lack of knowledge and skills in EBP among staff nurses.  Insufficient organizational support and resources for EBP.  The study found that for nurse leaders, only their EBP beliefs were significantly correlated to their implementation of EBP. Staff nurses had significantly lower EBP beliefs and implementation of EBP compared to nurse educators and leaders. | Recommendations:  Further research to explore the impact of specific educational interventions on EBP beliefs and implementation.  Strategies to enhance organizational readiness for EBP, including encouraging leadership support and resource allocation.  Provide ongoing education and training to improve nurses' knowledge and skills related to EBP. | Use of validated instruments to measure EBP-related variables.  Inclusion of a diverse sample of nurses in different roles (staff nurses, educators, leaders). | Conducted in only three hospitals, which may limit the generalizability of the findings.  Cross-sectional design, which limits the ability to infer causation. |
| (Caine & Kenrick, 1997)  UK | Qualitative exploratory study using semi-structured interviews with clinical directorate managers.  Thematic content analysis was used to analyse the interview transcripts. | The study included 10 directorate managers out of 29 eligible participants from two study sites (8 from site 1 and 2 from site 2).  Managers were required to have direct contact with and some degree of responsibility for their nursing team.  Participants varied in age, gender, educational background, and professional qualifications​ | The positive influence of NHS reforms that encouraged innovative thinking.  Performance reviews and appraisals which helped in identifying individual needs and setting motivational goals.  Organizational culture that supported the compliance of staff with research initiatives.  Managers saw their role as a facilitator, aiding nurses to overcome obstacles and ensuring organizational objectives, financial targets, and quality standards were met.  Managers used appraisals and performance reviews to encourage research utilization. | Conflicting demands on clinical managers and their inability to fully transition from nursing roles.  Limited time leading to the delegation of responsibilities to senior clinical practitioners.  Limited resources and dysfunctional budgetary allocations hindered NMs support for staff development and research utilization.  The perception that research utilization was an individual nurse's responsibility rather than a managerial one, resulting in managers abdicating their own responsibilities in creating a research-friendly environment.  Organizational constraints and managers' lack of interest in research-based clinical practice development. | Recommendation:  The authors recommend investing in initiatives that encourage collaboration between nurses and managers, such as performance reviews and appraisals, to promote research utilization. They also suggest that creating a research-friendly organizational culture is crucial for facilitating evidence-based practice​ | In-depth exploration of managers' perceptions and experiences through semi-structured interviews.  Inclusion of managers from multiple sites, providing a broader perspective.​ | Small sample size with less than half of the eligible managers interviewed (10 out of 29).  Potential biases due to the reliance on participants' willingness and availability.  Conducting the study in only two sites might not be representative of other settings |
| (Warren et al., 2016)  USA | Retrospective descriptive study using data from two online surveys conducted in 2008 and 2012.  The study focused on assessing the impact of previously implemented multifaceted interventions aimed at achieving and maintaining Magnet designation on the beliefs, readiness, and implementation of EBP among RNs and nurse leaders | Participants included nurse leaders and clinical RNs at the hospital.  2008: 275 nurses (213 clinical RNs, 62 nurse leaders).  2012: 339 nurses (250 clinical RNs, 89 nurse leaders) | Multifaceted interventions implemented to achieve and maintain Magnet designation.  Positive shift in clinical RNs' attitudes towards EBP and their perceptions of organizational readiness between the two survey years.  Positive learning environments.  Mentorship and addition of human and material resources  Educational programs and partnerships with schools of nursing. | Lack of time and heavy workloads.  Competing priorities and resistance to change.  Limited resources and support.  However, nurse leaders' EBPB and EBPI scores did not change significantly from 2008 to 2012, suggesting stagnation in their engagement with EBP. | Recommendations:  Leaders must recognize their role not only in facilitating EBP but also in actively engaging in and modelling evidence-based practices.  Leaders must set Realistic expectations and accessible resources for RNs.  A continued emphasis on leadership education and support. | Longitudinal comparison over a three-year period providing insight into changes over time.  Inclusion of both nurse leaders and clinical RNs allows for a comprehensive understanding of EBP beliefs, readiness, and implementation across different roles within the hospital.  Use of validated scales with high reliability. | The study relies on self-reported data, which can be subject to bias.  Specific details on the interventions and their exact nature are not provided in the extracted text, limiting the ability to assess their effectiveness fully.  Low response rates and small sample sizes2.  Potential biases due to turnover and economic factors. |
| (Shuman et al., 2019)  USA | Multisite cross-sectional descriptive study with data collection via electronic questionnaires | Participants included nurse managers (NMs) and registered nurses (RNs) from 24 adult medical-surgical units in seven community hospitals.  Nurse Managers (NMs): 24 invited, 23 responded  Staff Nurses (RNs): 553 invited, 287 responded2  Most nurse managers and staff nurses were Caucasian and female. Most nurse managers had a bachelor's or master's degree, while most staff nurses held a bachelor’s or associate degree. | Moderate extent in supportive and perseverant leadership  Somewhat competent in EBP knowledge and activities. | Low Proactive and Knowledgeable leadership  Modest unit climates for EBP with less-than-optimal practice climates for implementation of EBPs. Most importantly, rewarding EBP are relatively unsupported.  The study found that nurse managers' EBP competencies and leadership behaviours, as well as unit climates for EBP implementation, were modest at best. Both nurse managers and staff nurses perceived nurse managers' leadership behaviours and unit climates for EBP implementation as only moderately evident. | **Recommendation:**  Creating positive learning environments that promote knowledge and skill building, fostering a culture of inquiry, and providing mentorship and resources.  Ensuring continued leadership involvement and rewarding individual or team who has been instrumental in implementing the EBP.  Future studies investigate the interplay between social dynamic context factors and implementation strategies to promote the uptake of EBPs. | High Response Rates and high reliability of scales used for measuring EBP competencies and leadership behaviours. | Limited to specific hospital settings |
| (Chen et al., 2020)  China | Cross-sectional web-based survey | 1,017 nurse managers from 54 hospitals in Guangdong Province, China1 | Positive attitudes towards EBP  Supportive organizational culture  Receiving effective EBP education and training | Lack of EBP knowledge  Limited implementation of EBP  Organizational constraints such as lack of time and resources | Recommendations:  More attention needs to be paid on enhancing the EBP competence of nurse managers, as they play a unique role in driving evidence into practice. Suitable interventions and strategies such as providing effective education and training programs and creating a supportive organizational EBP culture and infrastructure should be developed and implemented. | Large sample size  Comprehensive analysis of factors influencing EBP | Cross-sectional design limits causal inferences  Self-report nature may introduce bias  Non-randomized sample limits generalizability |
