# Supplementary material for: Understanding, Using, and Facilitating Evidence-Based Practice: A Scoping Review of Influencing Factors Among Nurse Managers in Acute Care
Source: J Nurs Manag. 2025 Jul 4;2025:2155376. doi: 10.1155/jonm/2155376 (PMC12253997; doi:10.1155/jonm/2155376)
Supplement: Supporting Information 4 — Appendix 4: Quality appraisal of included studies. [file 2155376.f4.docx]

| **Study ID**  **Author(s)**  **Year of publication**  **Country of study** | **Abstract & Title** | **Introduction & Aims** | **Method & Data** | **Sampling** | **Data Analysis** | **Ethics & Bias** | **Results** | **Transferability** | **Implications & Usefulness** | **Total Score**  **(Out of 36)** |
| --- | --- | --- | --- | --- | --- | --- | --- | --- | --- | --- |
| (Gallagher-Ford et al., 2023)  United States | 4 | 4 | 3 | 3 | 4 | 4 | 4 | 3 | 4 | 33 |
| (Wilkinson et al., 2011)  Scotland, UK | 4 | 4 | 3 | 3 | 1 | 4 | 4 | 3 | 4 | 30 |
| (Camargo et al., 2018)  Brazil | 4 | 4 | 3 | 3 | 3 | 4 | 4 | 3 | 4 | 32 |
| (Camargo et al., 2016)  Brazil | 4 | 4 | 3 | 3 | 3 | 4 | 4 | 3 | 4 | 32 |
| (Shuman et al., 2018)  USA | 4 | 4 | 4 | 3 | 4 | 4 | 4 | 3 | 4 | 34 |
| (Lai et al., 2022)  China | 4 | 4 | 3 | 3 | 3 | 4 | 4 | 3 | 4 | 32 |
| (Kitson et al., 2011)  Australia | 4 | 4 | 3 | 3 | 3 | 4 | 4 | 3 | 4 | 32 |
| (Renolen et al., 2020)  Norway | 4 | 4 | 4 | 4 | 3 | 4 | 4 | 3 | 4 | 34 |
| (Weng et al., 2016)  Taiwan | 4 | 4 | 3 | 3 | 3 | 4 | 4 | 3 | 3 | 31 |
| (Royle et al., 1997)  Canada | 3 | 4 | 3 | 3 | 3 | 2 | 4 | 3 | 4 | 29 |
| (Johansson et al., 2010)  Sweden | 4 | 4 | 3 | 3 | 3 | 3 | 4 | 3 | 3 | 30 |
| (Kueny et al., 2015)  USA | 4 | 4 | 3 | 3 | 3 | 4 | 4 | 3 | 3 | 31 |
| (Barako et al., 2012)  Kenya | 4 | 4 | 3 | 3 | 3 | 4 | 4 | 3 | 4 | 32 |
| (Farokhzadian et al., 2015)  Iran | 4 | 4 | 3 | 3 | 3 | 4 | 4 | 3 | 4 | 32 |
| (Patton et al., 2024)  USA | 4 | 4 | 4 | 3 | 4 | 4 | 4 | 3 | 4 | 34 |
| (Hasanpoor et al., 2019)  Iran | 4 | 4 | 3 | 3 | 3 | 4 | 4 | 3 | 4 | 32 |
| (Almaskari, 2017)  Sultanate of Oman | 4 | 4 | 3 | 3 | 3 | 4 | 4 | 3 | 4 | 32 |
| (Lynn & Moore, 1997)  USA | 3 | 3 | 3 | 3 | 3 | 2 | 4 | 3 | 3 | 27 |
| (Mathew et al., 2024)  United States of America | 4 | 4 | 3 | 3 | 3 | 4 | 4 | 3 | 4 | 32 |
| (Gallagher-Ford, 2012)  USA | 4 | 4 | 3 | 3 | 3 | 4 | 4 | 3 | 4 | 32 |
| (Caine & Kenrick, 1997)  UK | 4 | 4 | 3 | 3 | 3 | 4 | 4 | 3 | 4 | 32 |
| (Warren et al., 2016)  USA | 4 | 4 | 3 | 3 | 3 | 4 | 4 | 3 | 4 | 32 |
| (Shuman et al., 2019)  USA | 4 | 4 | 3 | 3 | 3 | 4 | 4 | 3 | 4 | 32 |
| (Chen et al., 2020)  China | 4 | 4 | 3 | 3 | 3 | 4 | 4 | 3 | 4 | 32 |
