# Supplementary material for: Understanding, Using, and Facilitating Evidence-Based Practice: A Scoping Review of Influencing Factors Among Nurse Managers in Acute Care
Source: J Nurs Manag. 2025 Jul 4;2025:2155376. doi: 10.1155/jonm/2155376 (PMC12253997; doi:10.1155/jonm/2155376)
Supplement: Supporting Information 5 — Appendix 5: EBP Interventions for nurse mangers in acute care. [file 2155376.f5.docx]

### Appendix 5: EBP interventions for nurse managers in acute care

| Type of Intervention | Country | Income Level | Duration | Intervention purpose | Intervention Outcome |
| --- | --- | --- | --- | --- | --- |
| EBP Leadership Behaviour Program | USA | High | 12 hours | EBP leadership behaviours and competencies among nurse managers | Short-term improvement in leader self-reports; no change in leadership behaviour perceived by clinical nurses |
| Workshops guided by Rogers and Melnyk models | Brazil | Upper-Middle | Not specified (multiple sessions) | To build practical EBP skills and promote peer interaction among nurse managers | Short-term gains in motivation and understanding; long-term impact not assessed |
| University-accredited EBP course | Sweden | High | 5 days over 15 weeks | To improve head nurses’ ability to integrate EBP into managerial responsibilities | Improved head nurse engagement: long-term sustainability unclears |
| Knowledge Translation Toolkit with structured support | Australia | High | 12-month programme | To support clinical nursing leaders in implementing EBP through structured KT initiatives | Increased confidence and teamwork; long-term effects not measured |
| National multifaceted training and dissemination (TEBMA) | Taiwan | High | Multiple years (2007–2011) | To disseminate EBP knowledge and skills among Directors of Nursing through national strategies | Progressive reduction in barriers; long-term integration challenges remained |
| Workshops using hermeneutic-dialectic approach | Brazil | Upper-Middle | Five focus group sessions | To engage nurse managers in reflective practice and enhance openness to EBP through dialogue | Enhanced engagement and openness; long-term change not evaluated |
| Retrospective review of Magnet hospital interventions | USA | High | 4 years (2008–2012) | To evaluate the long-term organisational impact of Magnet designation on EBP adoption | Improved staff nurse EBP attitudes; no change in nurse leaders' EBP engagement |
